# A new tunable 3D alveolospheres model from human alveolar epithelial type 2 cells (AEC2) with reduced heterogeneity for studying cigarette smoke extract exposure

**DOI:** 10.1186/s12931-026-03628-z

**Published:** 2026-03-17

**Authors:** Marina Guecamburu, Arthur Pavot, Amélie Legrix, Caroline Jeannière, Yaniss Belaroussi, Matthieu Thumerel, Emma Samaniego, Hugues Begueret, Guillaume Maucort, Fanny Decoeur, Jean-William Dupuy, Anne-Aurélie Raymond, Pauline Esteves, Leo Grassion, Gael Dournes, Patrick Berger, Elise Maurat, Katharina Raasch, Eloïse Latouille, Vincent Studer, Isabelle Dupin, Pauline Henrot, Maéva Zysman

**Affiliations:** 1 Univ-Bordeaux, Centre de Recherche Cardio-thoracique de Bordeaux, U1045, CIC1401, Pessac, France; 2 INSERM, Centre de Recherche Cardio-thoracique de Bordeaux, U1045, CIC1401, Pessac, France; 3 Service de pneumologie, Service d’exploration fonctionnelle respiratoire, Service de réanimation, Service de chirurgie thoracique, CHU de Bordeaux, Service d’anatomopathologie, France; 4 Interdisciplinary Institute for Neuroscience, Centre National de la Recherche Scientifique, Bordeaux, France; 5 Interdisciplinary Institute for Neuroscience, University of Bordeaux, Bordeaux, France; 6 Université de Bordeaux, CNRS, INSERM, Bordeaux Imaging Center (BIC), US4, Bordeaux, 3420, 33000 UAR France; 7 France-BioImaging Core - UAR2057 CNRS UM, Bordeaux, France; 8 Univ. Bordeaux, CNRS, INSERM, TBM-Core, US5, UAR 3427, OncoProt, Bordeaux, F-33000 France; 9 Service des Maladies Respiratoires, CHU Bordeaux, Pessac, 33604 France

**Keywords:** Emphysema, 3-D organoid, Alveolar epithelial cells, Hydrogel microwells, Electron microscopy

## Abstract

**Rationale:**

Three-dimensional (3D) organoid models, such as alveolospheres, are unique tools for investigating the mechanisms underlying emphysema. However, high inter-organoid heterogeneity hampers consistent results in emphysema research and drug testing.

**Objectives:**

To develop a tunable 3D alveolosphere derived from human primary type II alveolar epithelial cells (AEC2) for modeling alterations linked to cigarette smoke exposure.

**Methods:**

AEC2 (HTII-280+) were isolated from 52 lung samples from both COPD and non-COPD patients, then cultured in 3D, comparing Matrigel to preformed photopolymerized hydrogel microwells of adjustable size and stiffness. Topological and phenotypic characterization were performed on days (D)1, 7, and 14. Lamellar bodies (LBs) were quantified using artificial intelligence (AI) analysis of transmission electron microscopy (TEM) serial block-face images. Chronic exposure to 1% or 5% cigarette smoke extract (CSE) was performed for 5 consecutive days.

**Results:**

Compared to Matrigel-based spheroid cultures, alveolospheres generated in microwells display reduced heterogeneity in size. Such alveolospheres were maintained in culture for 14 days and exhibited central lumen formation from D7 to D14. Across different hydrogel stiffness, a stiffness of 5 kPa was found to best support long-term organoid maintenance. The presence of tight junctions (TEM, ZO-1 immunostaining) suggested an auto-organization. AEC1 markers (P2XR4, PDPN) increased from D1 to D14 while AEC2 markers (ABCA3, SFTPA, SFTPC) persisted over time, in qPCR. TEM indicated surfactant synthesis, and AI-driven LB quantification revealed a decrease in LB-containing cells over time. CSE exposure resulted in cell death, architectural disorganization, oxidative stress, and inflammation. Similarly, alveolospheres derived from COPD patients showed increased expression of inflammatory and cell death markers.

**Conclusion:**

This standardized and adjustable 3D alveolosphere model, derived from human primary AEC2, successfully reproduces key native alveolar features. Exposure to CSE provides a relevant platform for studying responses to cigarette smoke exposure.

**Supplementary Information:**

The online version contains supplementary material available at 10.1186/s12931-026-03628-z.

## Background

Emphysema, a severe and globally prevalent respiratory disease and a major feature of chronic obstructive pulmonary disease (COPD), is primarily caused by long-term inhalation of particles such as those found in cigarette smoke. This exposure damages alveolar tissue, leading to breathlessness and reduced quality of life. Emphysema corresponds to the destruction of lung alveoli, resulting in airspace enlargement and decreased elastic recoil due to an imbalance of protease/antiprotease activity, increased apoptosis and oxidative stress, altering the mechanical forces experienced by alveolar epithelial and resulting in impaired lung repair [1]. Adult alveoli contain bipotent stem cells named alveolar epithelial cells type 2 (AEC2), which can self-renew and differentiate into surface-covering AEC1 cells.

Although animal models have contributed to our understanding of emphysema physiopathology, their translational relevance — particularly for modeling cigarette smoke-induced emphysema — remains limited as murine lungs differ anatomically (notably lacking respiratory bronchioles) and human AEC2s exhibit distinct markers and smoke-induced responses compared with their rodent counterparts [2]. This highlights a critical need for innovative in vitro models based on human cells to better reflect COPD physiopathology. Three-dimensional (3D) organoids, which are self-assembled cultures derived from pluripotent stem cells (PSCs) or adult progenitors, are unique tools for modeling various organ functions [3, 4]. In the context of lung research, alveolospheres are organoids used to model alveolar functions [5] such as barrier function and surfactant production—both of which are impaired in emphysema [6, 7]. However, current alveolospheres, generated by embedding AEC2 in Matrigel™ [5], exhibit heterogeneity in size, shape, and cellular composition, which compromises robustness and limits their utility in understanding disease development and progression. Reduction of this physical variability [8, 9] and replication of in vivo alveolar anatomy, including lumen formation, remain major challenges [10].

Microstructured hydrogels offer a precise way to control organoid size [11], but because they are often molded on commercial surfaces, the ability to adjust microwell size and substrate rigidity is limited. Therefore, the ability to tailor hydrogel structural properties—combining spatially tunable sizes and stiffness—would provide an unprecedented tool to mimic features relevant for emphysema pathophysiology, such as the mechanical microenvironment of emphysematous lung tissue.

In this study, we aimed to form homogeneous alveolospheres by seeding human primary AEC2 (HTII-280⁺) into microwells with standardized size and stiffness, created using PRIMO technology [12]. The models demonstrated progressive lumen formation and the presence of AEC2 and evidences of AEC2 differentiation towards an AEC1 phenotype, as determined using innovative transmission electron microscopy (TEM) and AI-driven image quantification. Moreover, to model cigarette-smoke-induced alterations, we used cigarette smoke extract (CSE); although the primary insult in emphysema is airborne, soluble components provide a valid method for assessing epithelial-specific toxicity in submerged 3D culture. In our model, chronic CSE exposure increased oxidative stress, inflammation, and altered cell junction’s integrity. Finally, alveolospheres formed with cells derived from COPD patients retained characteristic features of emphysema, demonstrating the relevance of our model to study associated pathophysiology.

## Methods

### Microwell production

**Fig. 1:**
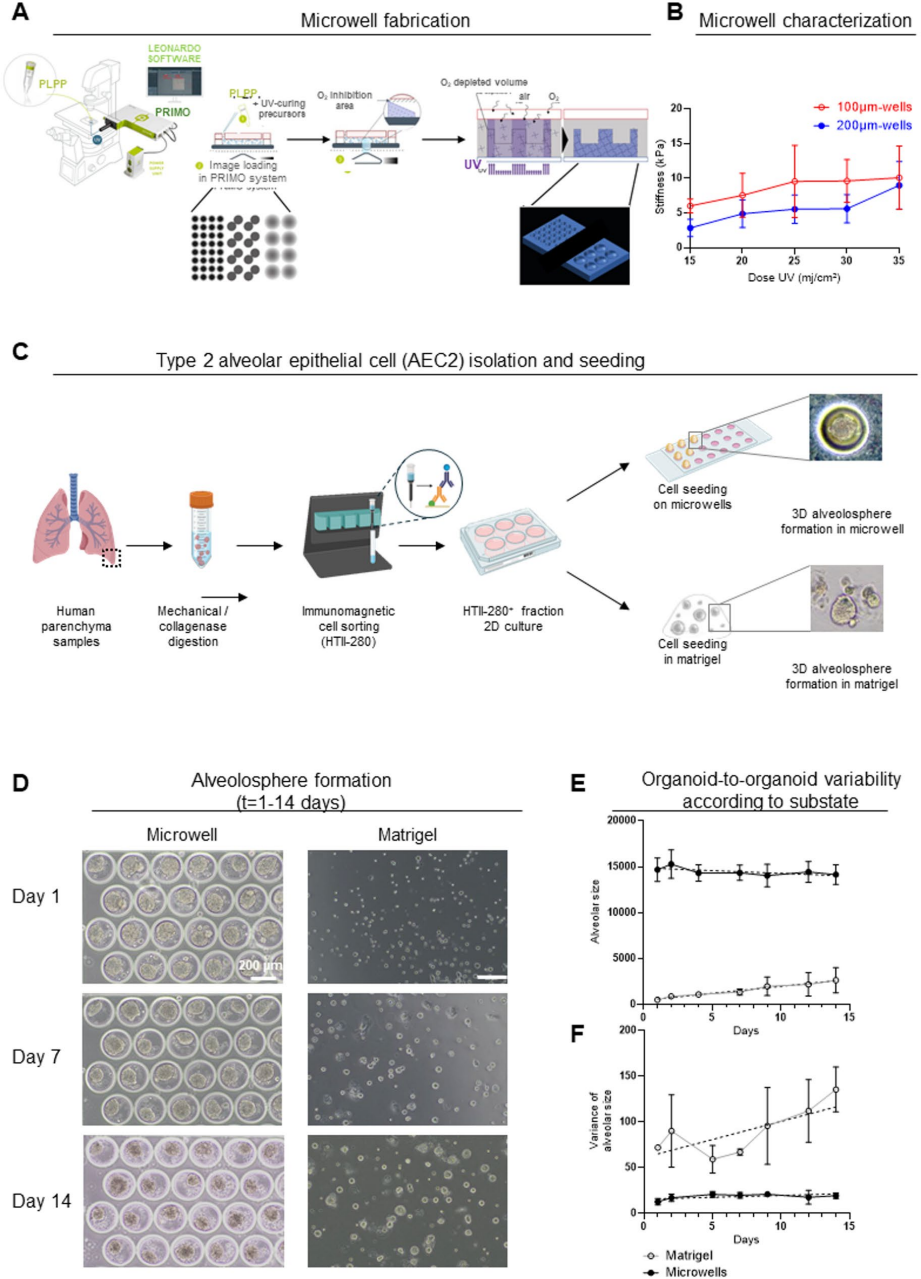
Formation of 3D alveolospheres from primary human type 2 alveolar epithelial cells, Matrigel-based versus microwells. **A** Formation of hydrogel microwells by UV transillumination. The light-based system creates microwells with adjustable shape, size, and stiffness in the hydrogel, utilizing a photoinitiator solution (PLPP, benzophenone gel) and its interaction with oxygen. **B** Microwell stiffness, measured by Chiaro Optics 11, for 100-µm- and 200-µm-diameter wells (*n* = 3 technical replicates). **C** Isolation and culture of type 2 alveolar epithelial cells (AEC2) from human lung samples in microwells of different sizes and shapes for alveolosphere formation. AEC2 were purified using HTII-280 immunomagnetic-activated cell-sorting beads. **D** Representative bright-field images of AEC2 seeded into microwells as compared to Matrigel, over 14 days. **E** Compared to Matrigel-based alveolospheres cultures (grey), alveolospheres generated in microwells (black) remained stable in size over time (*n* = 3 patients). **F** Compared to Matrigel-based alveolospheres cultures (grey), alveolospheres generated in microwells (black) display reduced variance in size (*n* = 3 patients)

Microwells were manufactured using the PRIMO system (Alvéole™), as previously described [12]. Briefly, four 3 × 3 mm polydimethylsiloxane (PDMS) microwells (Alvéole Stencell) were placed on a 35-mm-diameter Ibidi glass-bottom Petri dish. A mixture of polyethylene glycol acrylate powder (4ARM-PEG-AVRL-10 K, a semi-rigid hydrogel) and photoinitiator solution (PLPP, benzophenone gel) in phosphate-buffered saline was added to each PDMS microchamber. Patterned ultraviolet (UV) irradiation (between 20 and 35 mJ/mm²) using a digital micromirror device (Alvéole PRIMO) was applied to induce hydrogel photopolymerization, resulting in tailor-made microwells (54 microwells per microplate) to achieve stiffness levels mimicking those observed in native lung tissue (e.g., 5 kPa) [13–15]. (Fig. 1A). Hydrogel stiffness was assessed using microindentation characteristics (Chiaro, Optics11, Fig. 1B).

### Lung tissue processing

Tumor-free lung tissue, at least 2 cm away from the tumor, was obtained from patients undergoing surgery for lung cancer (smokers, ex-smokers, and non-smokers) or from emphysematous lung explants after transplantation at the Bordeaux University Medical Center (France) (Supplementary Figure S1). COPD patients were defined as individuals exhibiting non-reversible airflow obstruction, characterized by a forced expiratory volume in 1 s (FEV1) / forced vital capacity (FVC) ratio < 0.7 post bronchodilation [16]. Patient characteristics are summarized in Table 1.

**Table 1:** Patients characteristics

	*N* = 52
Age, years	65 [58; 71]
Sex (female, male)	27 (52)
BMI, kg/m^2^	25.3 [22.5; 27.0]
Smoking, pack-years	29 [10; 50]
Smoking habits	
Never smoker Former smoker Current smoker	9 (17) 36 (69) 7 (13)
FEV1/FVC < 0.7	6 (12) ^a^
FEV1/FVC	0.73 [0.72; 0.82] ^a^
FEV1, L	2.21 [1.80; 2.71] ^a^
FEV1, % predicted	86 [73; 108] ^a^
FVC, L	2.98 [2.28; 3.63] ^a^
RV, L	2.46 [1.78; 2.82] ^b^
RV, % predicted	119 [90; 130] ^b^
TLC, L	6.46 [4.73; 6.57] ^b^
TLC, % predicted	100 [86; 113] ^b^
DLCO, %	67 [54; 86] ^c^
PaO_2,_ mmHg	74 [71; 88] ^d^
pH	7.43 [7.41; 7.46] ^d^
Emphysema (LAA% <950 HU)	4 (8) ^e^
Cancer	
Adenocarcinoma Squamous cell cancer Other	31 (60) 5 (10) 7 (13)
Transplantation	
Emphysema COPD lung explants	4 (8) 5 (10)
Lung samples	
Weight (g) 2D culture (days) Total number of cells (.10^6^) Number of HTII-280 + cells (.10^6^)	2.8 [1.4; 3.9] ^f^ 25 [20; 27] ^g^ 308 [107; 401] ^h^ 3.9 [1.15; 3.95] ^i^

Missing values are as follows: ^a^ *n* = 3, ^b^ *n* = 6, ^c^ *n* = 15, ^d^ *n* = 28, ^e^ *n* = 13, ^f^ *n* = 4, ^g^ *n* = 3, ^h^ *n* = 8, ^i^ *n* = 4

*2D* two-dimensional, *BMI* body mass index, *DLCO* diffusing capacity of carbon monoxide in the lung, *FEV1* forced expiratory volume in 1 s, *FVC* forced vital capacity, *HU* Hounsfield units, *PaO*_*2*_ partial pressure of oxygen in arterial blood, *RV* residual volume, *TLC* total lung capacity. Low attenuation area (LAA%) was defined as the percent of voxels below − 950 HU. Data are presented as n (%) or median [quartile 1; quartile 3]

### Quantification of emphysema via computed tomography scans

Chest CT-scans (GE Revolution) were used to quantify emphysema (Supplementary Methods and Supplementary Figure S2). Briefly, emphysema was defined for this analysis as the proportion of voxels within lung tissue that exhibited an attenuation value lower than − 950 Hounsfield units (LAA-950HU), a threshold considered indicative of clinically significant emphysema when exceeding 6% [17].

### AEC2 selection and culture

AEC2 were isolated through immunomagnetic cell sorting based on the surface expression of the 280-kDa human type II cell protein (HTII-280, Terrace Biotech; Supplementary Methods). Isolated AEC2 were initially expanded in two-dimensional (2D) six-well plates (500,000 cells/well seeding density), as previously published [5], in Pneumacult ExPlus culture medium (Stemcell Technologies). After 5 to 7 days, the medium was switched to Pneumacult Ex Basal culture medium (Stemcell Technologies), and AEC2 were further amplified 6fold, until reaching 70–90% confluency (Fig. 1C).

### AEC2 seeding in 3D microwells

AEC2 cells were incubated with 0.05% trypsin (Trypsin-EDTA Solution, Gibco) and reseeded either in Matrigel (BME, cultrex type 1, 500 cells/µl) or in microwells (10,000 cells/µL) both in Pneumacult Airway Organoid Basal Medium (Stemcell Technologies, Fig. 1C). Forty-eight hours later, the culture medium was replaced with organoid differentiation medium (Stemcell Technologies). Thereafter, the medium was changed every 3 days; alveolospheres were photographed daily using a brightfield inverted microscope (Olympus) until day 14.

### Transmission electron microscopy

The 3D alveolospheres were analyzed using Serial Block-Face TEM (SBF-TEM) and serial scanning electron microscopy (as detailed in the Supplementary Methods, Supplementary Figure S3). By analyzing an entire organoid through serial imaging and sectioning, volumes were reconstructed to reveal the intricate 3D architecture of organelles within the cells. Lumen volume was quantified for all organoids fixed in MET, after reconstruction of an entire organoid via serial block-face scanning electron microscopy (SBF-SEM), across culture days 1 to 14.

### Artificial intelligence–driven structure prediction for lamellar bodies in SBF-TEM

We developed an artificial intelligence (AI)–driven model to predict lamellar bodies (LBs) in whole organoid samples. For automated cell segmentation, the model was trained using previously annotated datasets. Briefly, after cell segmentation via Cellpose, morphological features including nuclei and LBs were extracted using Ilastik. AI-driven quantification was then used to assess structural changes over time (Supplementary Methods and Supplementary Figure S3), specifically the proportion of cells containing LBs (regarded as AEC2) relative to those without LBs (regarded as AEC1).

### CSE exposure

A 100% (undiluted) CSE was generated by passing the smoke from two research-grade filtered cigarettes (Kentucky Tobacco Research and Development Center, University of Kentucky, Lexington, KY, USA) through 20 mL of Stable Cell DMEM–high glucose medium (Sigma-Aldrich) using a disposable tube and a manual syringe. To ensure consistency, all experiments were performed using a single CSE batch prepared on the same day, which was aliquoted and stored at − 80 °C, so that 100% CSE was identical across all experimental runs. Then, it was thawed and diluted to 1% or 5% with Organoid Differentiation Culture Medium accordingly. Alveolospheres from non-COPD patients were exposed to CSE (with daily medium replacement) for 5 consecutive days beginning on day 7 of culture. Cell viability after 5 days of exposure was qualitatively assessed via calcein labeling (Calcein AM, Thermo Fisher, C3099).

### Label-free quantitative proteomics

One dish (54 alveolospheres from the same patient per dish) were pooled to obtain sufficient material. Cell lysis was performed using RIPA buffer. Each lysate was centrifuged, and the supernatant was used for proteomic analysis (Supplementary Methods). Mass spectrometry proteomics data were deposited with the ProteomeXchange Consortium via the PRIDE (X3) partner repository [18]; the dataset identifier is available upon reasonable request.

### Ethics and statistics

Surplus lung tissue obtained after surgery was used for research within the context of patient care, under a no-objection system for the coded anonymous use of residual diagnostic or therapeutic tissue. This non-interventional research protocol received approval from the Bordeaux University Hospital’s TUBE agreement, version 1 (CHU BX 2020/54, 14/01/2021), and was performed in accordance with the Declaration of Helsinki.

Continuous variables were compared using non-parametric tests (Wilcoxon, Kruskal–Wallis, Spearman) and are presented as means ± standard deviations or medians [Q1–Q3]. Individual data points with standard deviations are shown in figures. Data are presented as mean ± SEM from *n* = 3 to 8 biological replicates, accordingly. Statistical significance was assessed using non-parametric tests, or non-parametric paired-t-tests when appropriate, with significance defined as *p* < 0.05. Statistical significance across multi-comparison test was evaluated using two-way ANOVA, followed by Tukey’s post-hoc test for pairwise comparison. Statistical analyses were performed using GraphPad Prism version 10 (GraphPad Software Inc., La Jolla, CA, USA), and images were analyzed using ImageJ/ImageLab.

## Results

### Standardized alveolospheres from human primary AEC2

Seventy-two lung samples from COPD and non-COPD subjects were collected between 1 February 2023 and 31 July 2024. After the exclusion of 20 samples due to insufficient initial AEC2 amplification in 2D cultures (*n* = 14), lack of cellular adhesion to the hydrogel (*n* = 3), or contamination (*n* = 3), 52 lung samples were included in the analysis (Supplementary Figure S1). The median weight of parenchymal tissue was 2.8 g, and the median duration of the 2D amplification phase for AEC2 was 25 days. Subjects characteristics are summarized in Table 1. Briefly, 52% of patients were women, with a median age of 65 years; 36 (69%) were former smokers with a median smoking history of 29 pack-years. Six (12%) patients exhibited airflow obstruction, and four (8%) had emphysema.

There were no significant associations between the number of purified AEC2 per lung sample or between the duration of the 2D culture phase and airflow obstruction, or between the number of isolated AEC2 cells per gram of lung tissue and smoking history or age. However, AEC2 cell amplification in 2D culture failed more frequently when cells were collected from patients with more severe airflow obstruction (Supplementary Table S1).

HTII-280 cell sorting enriched epithelial cells in the positive fraction; no epithelial cells were detected in the negative fraction by FACS (Supplementary Figure S4 A, B, C). Cell viability remained high during purification, except immediately after sorting in the positive fraction (Supplementary Figure S4 D). The 2D culture step amplified viable epithelial cells and eliminated contaminating CD45⁺ cells from the positive fraction: before seeding into microwells, 96% of the population was 4′,6-diamidino-2-phenylindole (DAPI)–negative and 84% was pan-cytokeratin–positive (Supplementary Figure S4 B). After expansion (20 ± 5 days) in 2D culture, cells expressed epithelial markers (pan-cytokeratin) and lacked hematopoietic markers (CD45, Supplementary Figure S4 E, F, G). FACS analysis indicated a high proportion of cells positive for epithelial cell adhesion molecule (EpCAM) (Supplementary Figure S5 A) consistent with the strong E-cadherin staining observed (Supplementary Figure S5 B). Moreover, these cells were predominantly AEC2, expressing surfactant protein C (SPC) and positive for Lysotracker staining while negative for podoplanin (PDPN), HTI-56 (human type I cell), keratin 5 (KRT-5) and secretoglobin 1A1 (SCGB1A1) staining, meaning that there was no switch towards AEC1 (Supplementary Figure S5 C, D). Of note, the HT2-280 negative fraction was also characterized and contain a small proportion of bronchiole epithelial cells assessed by FACS analysis with 22% of CK5⁺ cells (Supplementary Figure S5 E).

In order to assess the ability of microwells to lead to alveolosphere formation in comparison with the standard culture in Matrigel, we seeded AEC2 from 3 different subjects in both conditions (Fig. 1D). Alveolospheres seeded into Matrigel were initially smaller, and increased moderately in size over time, whereas those seeded in microwells remained stable from day 1 to 14 (Fig. 1E). Compared to Matrigel-based spheroid cultures, alveolospheres generated in microwells display reduced heterogeneity in size, assessed through the evolution of variance along time (Fig. 1F). Therefore, organoid-to-organoid variability was minimized through standardized microwells conditions.

**Fig. 2:**
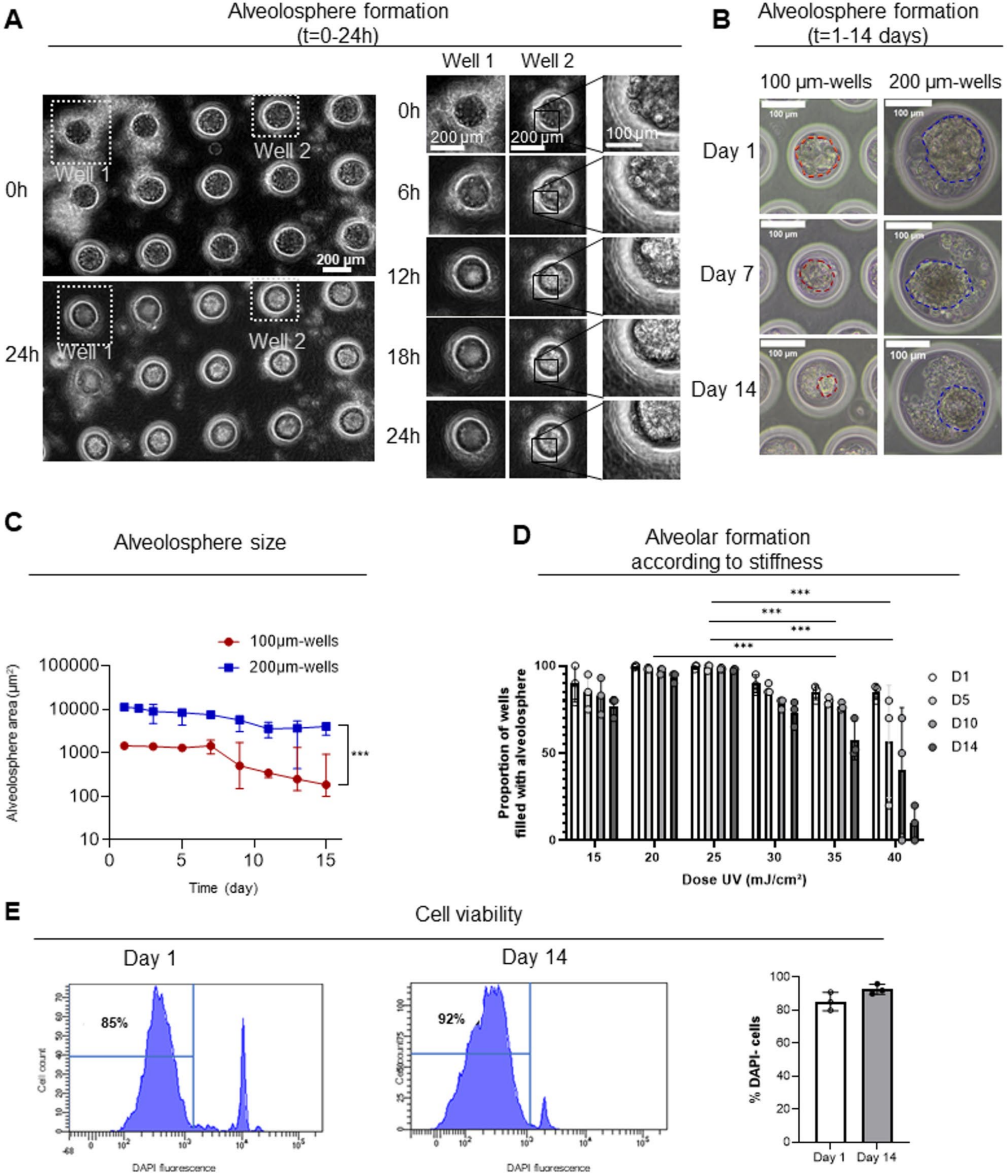
Formation of 3D alveolospheres from primary human type 2 alveolar epithelial cells. **A** Bright-field live imaging of AEC2 seeded into microwells during the first 24 h. Left panels: representative images at the start (0 h) and 24 h after cell seeding. Right panels: magnified views of Well 1 and Well 2 (indicated in the left panel). **B** Representative bright-field images of AEC2 seeded into microwells of different sizes (diameters: 100 μm and 200 μm) over 14 days. Dotted lines outline the alveolospheres. **C** Quantification of alveolosphere areas over time in microwells of 100 μm (n = 3 patients) and 200 μm (n = 5 patients) in diameter. **D** Number of alveolospheres over time in microwells of different stiffnesses, depending on UV exposure (n = 3 patients) (**E**) Viability of alveolospheres grown in 200-µm-diameter microwells, quantified by flow cytometry. Left panel: histograms showing representative cell counts (y-axis) versus DAPI fluorescence (x-axis) on day (D) 1 and D14 after AEC2 seeding. Percentages indicate proportions of DAPI-negative (viable) cells. Right panel: percentage of DAPI-negative cells on D1 and D14 after AEC2 seeding (n = 3 patients). Data are presented as mean ± SEM from n = 3 to 5 biological replicates as mentioned. Statistical significance was assessed using non-parametric tests, ** p = 0.001, *** p = 0.001. 2D: two-dimensional; 3D: three-dimensional; AEC2: alveolar epithelial cell type 2; HTII-280: human type 2 cell marker; t: time

After being seeded in microwells, AEC2 self-organized into spherical structures within the first 24 h. Time-course analysis allowed visualization of the gradual self-organization of alveolospheres (Fig. 2A). Those cultured for 14 days showed a 72% overall success rate in terms of alveolar organoid formation (Supplementary Figure S1). We evaluated the impact of microwell diameter on alveolospheres formation. The diameters of the alveolospheres were greater in the 200-µm-diameter wells than in 100-, 150- or 250-µm-diameter wells (Fig. 2B, C, Supplementary Figure S6). Thanks to the modular nature of the hydrogel, we also evaluated the impact of different hydrogel stiffnesses on alveolospheres longevity. Two-way ANOVA revealed a significant effect of stiffness on alveolosphere formation (F(df1, df2) = 5.12, *p* = 0.0003). Post-hoc comparisons showed that the 5 kPa stiffness, corresponding to 20–25 mJ/cm² UV exposure during cross-linking conditions differed significantly from the 10 kPa, corresponding to 35–40 mJ/cm² conditions (Fig. 2D). Finally, according to flow cytometry analyses, the model showed a mean viability of 85% of live cells on day 1 (D1) and 92% on D14 (Fig. 2E). In light of these results, we selected 5 kPa and 200-µm-diameter microwells for subsequent experiments. Very interestingly, they were in accordance with physiological parameters of native lung tissue (~ 5 kPa stiffness [13]) and alveolar size (~ 200 μm in diameter [19]).

### Model characterization: topology

**Fig. 3:**
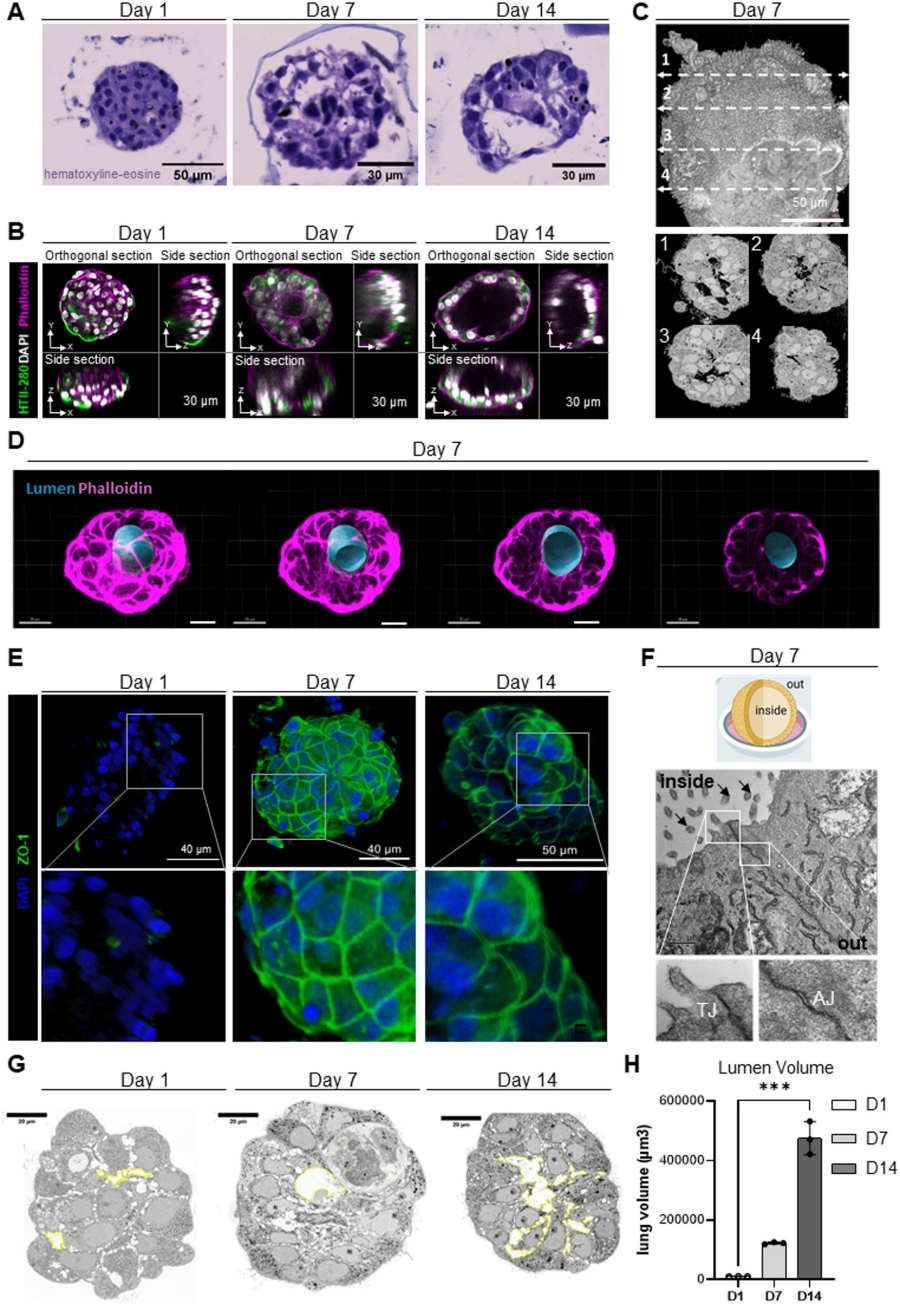
Topology of the 3D alveolosphere model: lumen formation, barrier function, and polarity. **A** Representative bright-field images of hematoxylin–eosin (HE)-stained sections on day (**D**) 1, D7, and D14. **B** Fluorescent images of alveolospheres stained for F-actin (phalloidin, magenta), the type 2 alveolar epithelial (AEC2) cell marker (HTII-280, green), and nuclei (DAPI, white), showing progressive lumen formation and persistent AEC2 cells on D1, D7, and D14. **C** Reconstruction of a complete alveolosphere on D7, revealing a central lumen obtained from Transmission Electron microscopy. **D** Transverse sections of a three-dimensional (3D) alveolosphere on D7, obtained from Z-stack spinning disk images and stained for F-actin (phalloidin, magenta), with lumen reconstruction (blue). **E** 3D reconstruction of an alveolosphere stained for zona occludens-1 (ZO-1, green) and nuclei (DAPI, blue), imaged by spinning disk microscopy on D1, D7, and D14, showing progressive tight junction (TJ) formation. **F** Top panel: schematic of an alveolosphere grown in a microwell with an inside-out orientation (created with BioRender.com). Bottom panels: electron microscopic image of an embedded alveolosphere on D7, showing intercellular adhesion with tight junctions (TJs) and adherens junctions (AJs). Arrows indicate microvilli. **G** Electron microscopic image of an embedded organoid on day (D) 1, 7 and 14. The lumen volume was measured (yellow line). **H** Quantification of the lumen volume, after reconstruction of an entire organoid via serial block-face scanning electron microscopy (SBF-SEM), from 3 patients at day (D) 1, 7 and 14. Data are presented as mean ± SEM. Statistical significance was assessed using non-parametric tests, *** *p* = 0.001

The progressive formation of a lumen, confirmed by hematoxylin–eosin (HE) staining of organoid sections, 3D confocal imaging of whole structures, and TEM imaging, was a key feature demonstrating ability of AEC2 cells to self-organize into alveoli-like 3D models (alveolospheres) (Fig. 3A-C). Indeed, HE staining of paraffin-embedded sections and TEM imaging revealed several internal cavities around D7, which merged into a single central lumen by D14, resembling the architecture found in vivo (Fig. 3C). Confocal 3D microscopy further confirmed the presence of an internal lumen (Fig. 3D). Next, we evaluated cellular junction with staining for the tight junction marker zona occludens-1 (ZO-1) (Fig. 3E) and ultrastructural characterization by electron microscopy. AEC2 became cohesive and adherent over time, and we observed tight and *adherens* junctions via both ZO-1 staining and TEM imaging on D7 (Fig. 3E, F). The tight junctions and microvilli were located at the apical part of the AEC2 cells lining the lumen, suggesting formation of a partially polarized epithelium. The lumen volume was calculated relative to the total organoid volume, providing an estimate of the proportion of the organoid occupied by the lumen (Fig. 3G). Lumen volumes ranged from 10,665+/-442 μm³ at day 1 to 474,592+/-55,107 μm³ at day 14 (Fig. 3H), while total organoid volumes ranged from 931,985 +/-122,501 μm³ at D1 to 1,108,800 +/-253,905 μm³ at D14. The resulting lumen-to-organoid volume ratios increased from 1% at D1 to 42% at D14. These measurements indicate that lumen formation was generally observed across organoids, with significant increasing proportional volume over culture time, although some variability exists between individual organoids.

### Characterization of cellular differentiation

**Fig. 4:**
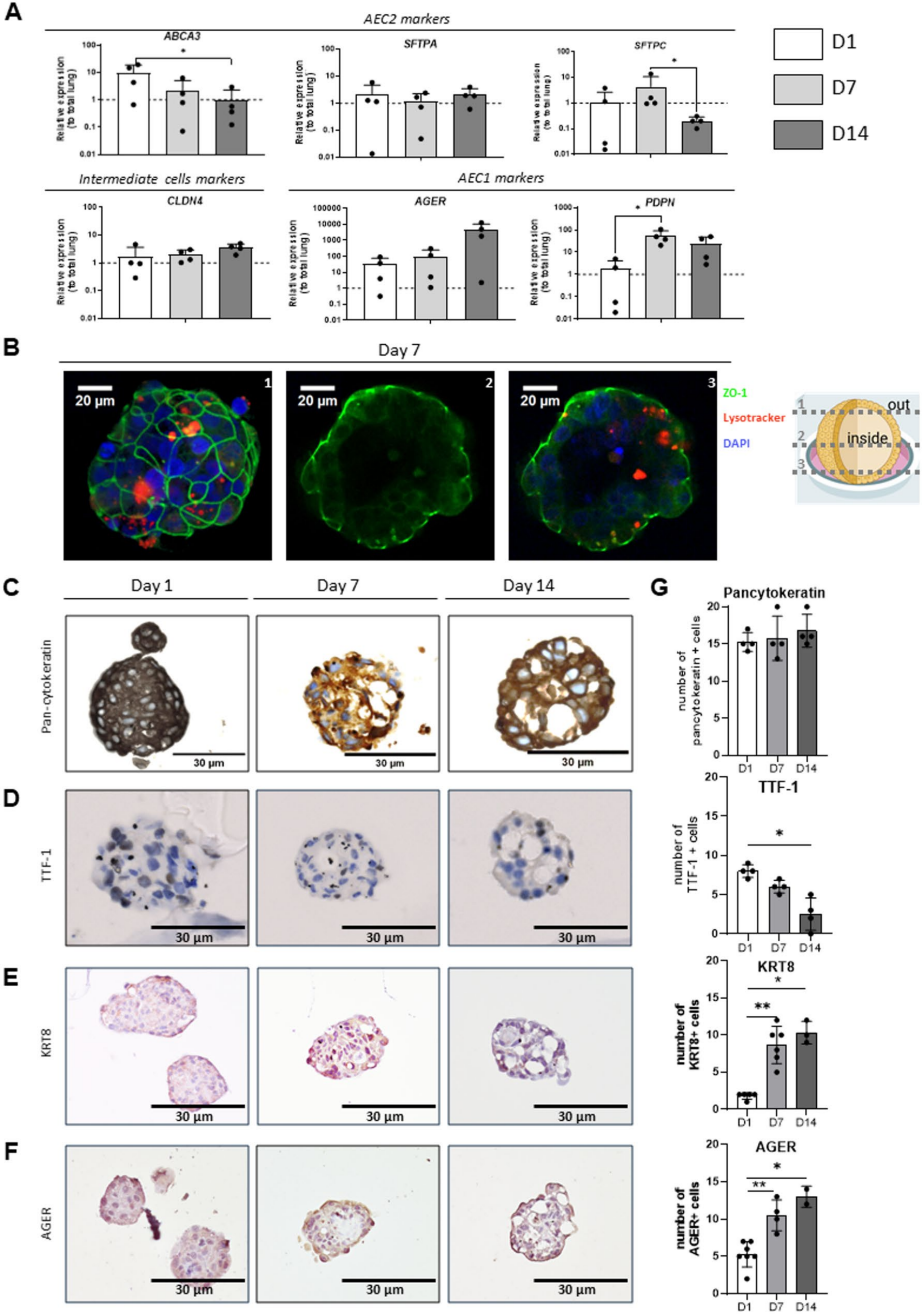
Characterization of cellular differentiation in the 3D alveolosphere model. **A** qPCR results showing the persistence of AEC2 gene expression (*ABCA3*, *SFTPA*, *SFTPC*) over time; markers of intermediate cells (*CLDN4*) and AEC1 cells (*AGER*, *PDPN*) progressively increased in four different patients. Statistical significance was assessed using non-parametric tests, * *p* = 0.05. **B** The presence of surfactant-producing organelles was evidenced by Lysotracker, in red staining at day 7. The images represent different sectional planes within the same alveolosphere, illustrating the 3D organization. **C** Bright-field images of HE staining and pancytokeratin immunostaining. One representative paraffin-embedded tissue section was assessed per donor for each staining. **D** Bright-field images of HE staining and TTF-1. One representative paraffin-embedded tissue section was assessed per donor for each staining. **E** Bright-field images of HE staining and KRT8 immunostaining. One representative paraffin-embedded tissue section was assessed per donor for each staining. **F** Bright-field images of HE staining and AGER immunostaining. One representative paraffin-embedded tissue section was assessed per donor for each staining. **G** Quantification from 4 to 6 patients at day (**D**) 1, 7 and 14, for pancytokeratin, TTF-1, KRT8 and AGER. Data are presented as mean ± SEM. Statistical significance was assessed using non-parametric tests, * *p* = 0.05, ** *p* = 0.01. *ABCA3*: ATP-binding cassette subfamily A member 3; AEC1: alveolar type 1 cell; AEC2: alveolar type 2 cell; AGER: Advanced Glycosylation End-Product–specific Receptor, *CLDN4*: claudin-4; HTII: human type 2 cells; KRT8: Keratin 8; *P2 × 4R*: purinergic receptor P2 × 4; *PDPN*: podoplanin; *SFTPA*: surfactant protein A; *SFTPC*: surfactant protein C; TTF-1: thyroid transcription factor 1; ZO-1: zona occludens-1

Alveolospheres show proliferative activity, as confirmed by Ki-67 staining at D1, D7 and D14, indicating ongoing even decreasing cell division within the structures (Supplemental Figure S7 A, B). Phenotypic characterization over time using quantitative polymerase chain reaction [qPCR] showed progressive decreased expression of AEC2 marker genes (ABCA3, SFTPA, SFTPC), whereas markers of intermediate cells (CLDN4) and AEC1 cells (P2 × 4R, PDPN) progressively increased (Supplemental Table S2, Fig. 4A). The presence of surfactant-producing organelles was evidenced by Lysotracker staining at day 7 (Fig. 4B), which accumulates in lamellar bodies (LBs) [20]. Pan-cytokeratin staining confirmed the presence of epithelial cells and remained stable over time (Fig. 4C). HTII-280 immunostaining (Fig. 3B), and the number of thyroid transcription factor-1 (TTF-1, Fig. 4D) surfactant protein C and (SFTPC, Supplementary Figure S7 E, F) positive cells, for AEC2, significantly decreased from D1 to D14. We also confirmed the increasing number of transitional cells, expressing keratin 8 (KRT-8, Fig. 4E) and the presence of SCGB3A2 + cells (Supplementary Figure S7 C, D), associated with an increasing number of AEC1 assessed by AGER staining (Fig. 4F). Altogether these findings show the presence and maintenance of a pool of AEC2 overtime, together with their differentiation towards an AEC1 phenotype.

### Electron microscopy characterization

**Fig. 5:**
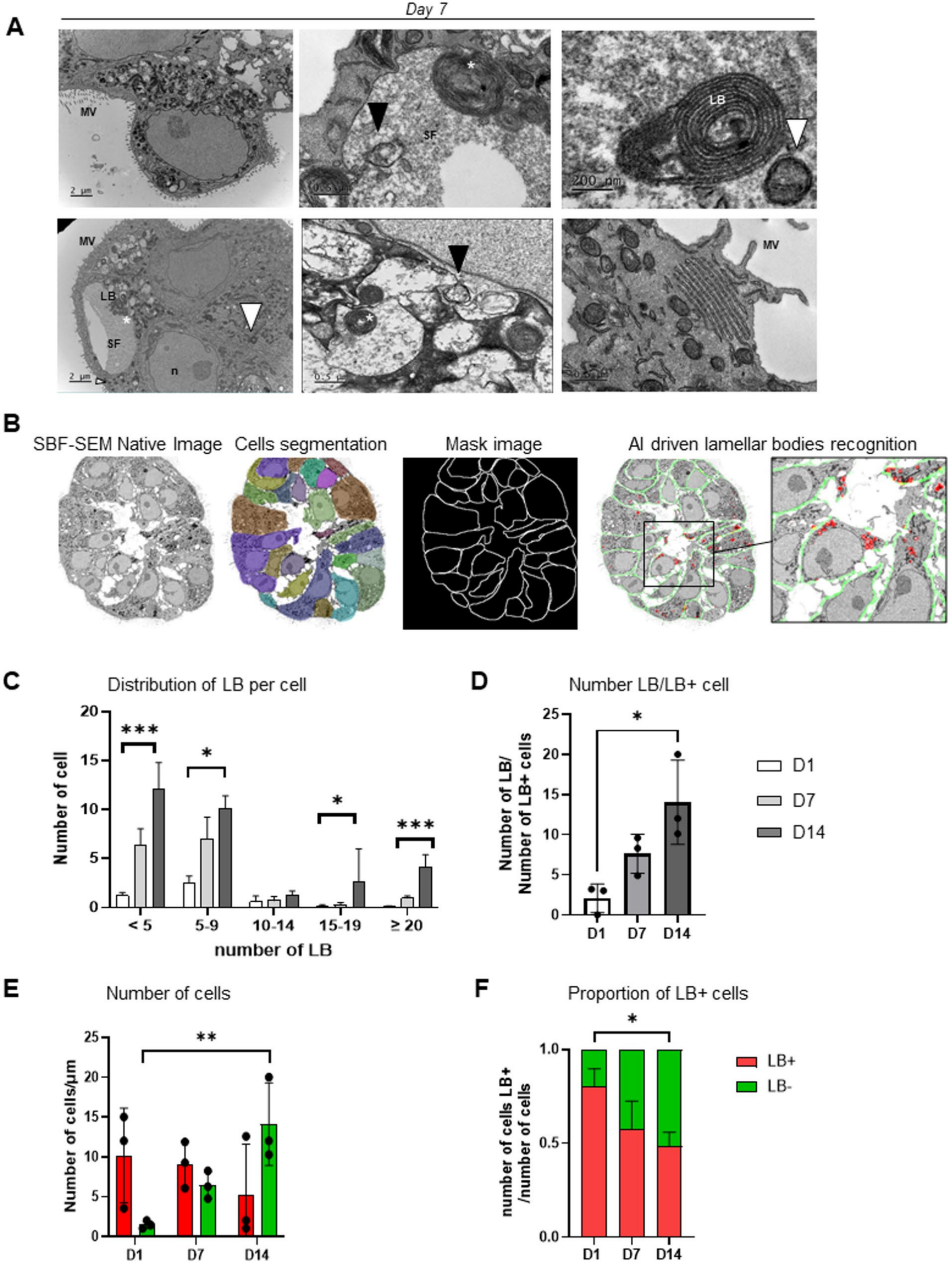
Characterization of cellular differentiation in the 3D alveolosphere model by electron microscopy. **A** Electron microscopic image of an embedded organoid on day (**D**) 7. LB: lamellar body; SF: surfactant; MV: microvillosity; n: nucleus. White asterisks indicate extracellular LBs; black arrow indicates an uncoiled extracellular LB; white arrows indicate lipid bodies. **B** Reconstruction of an entire organoid via serial block-face scanning electron microscopy (SBF-SEM), automatic cellular segmentation, and automatic detection of LBs. **C** Distribution of LBs per cell in organoids on D1, D7, and D14, *n* = 3 patients. **D** Number of LBs per LB-containing cell in organoids on D1, D7, and D14, *n* = 3 patients. **E** Quantification of the number of cells with (red) and without (green) LBs in organoids on D1, D7, and D14, *n* = 3 patients. **F** Proportion of LB-containing cells (red) relative to cells without LBs (green) in organoids on D1, D7, and D14, *n* = 3 patients.Data are presented as mean ± SEM. Statistical significance was assessed using non-parametric tests, * *p* = 0.05. ** *p* = 0.01, *** *p* = 0.001. AEC2: alveolar type 2 cell; AI: artificial intelligence; LB: lamellar body; MV: microvillosity; n: nucleus; PDPN: podoplanin; SBF-SEM: serial block-face scanning electron microscopy; SF: surfactant

Electron microscopy analyses revealed abundant mitochondria and numerous LBs, characteristics of mature AEC2 cells (Fig. 5A). Notably, the mean diameter of LBs was approximately 600 nm, consistent with previous reports [21]. We also identified different maturation stages of LBs (intracellular, extracellular coiled, and uncoiled), as well as numerous lipid bodies, suggesting active surfactant production and secretion. Additionally, we observed isolated, connected, and membrane-attached LBs, as previously described [21]. To precisely assess LB presence and quantity over time, we developed an AI-based tool for their detection and quantification using whole TEM images. These images were previously segmented to delineate cell membranes (Fig. 5B and Supplementary Figure S2) and then reconstructed into 3D images using SBF-TEM. We found that the overall number of LBs increased over time (Fig. 5C), along with the number of LBs per LB-containing cell, meaning that the concentration of LBs increased in specific cells (e.g., AEC2, Fig. 5D). Concurrently, the total number of LB+ cells per alveolosphere decreased, and the number of LB- cells increased (e.g., intermediate cells or AEC1, Fig. 5E). Moreover, the proportion of LB-containing cells (e.g., AEC2) relative to those without LBs (e.g., intermediate cells or AEC1) significantly decreased over time (Fig. 5F). Collectively, these data suggest the progressive differentiation and maturation of AEC2 cells into intermediate and possibly AEC1 cells within the alveolospheres.

Taken together, our 3D alveolosphere model meets established organoid criteria [4], including the presence of multiple organ-specific cell types, the recapitulation of key organ features such as lumen formation and cellular junction, and the presence of LB-containing AEC2 cells and differentiated intermediate cells.

### Chronic exposure to cigarette smoke

**Fig. 6:**
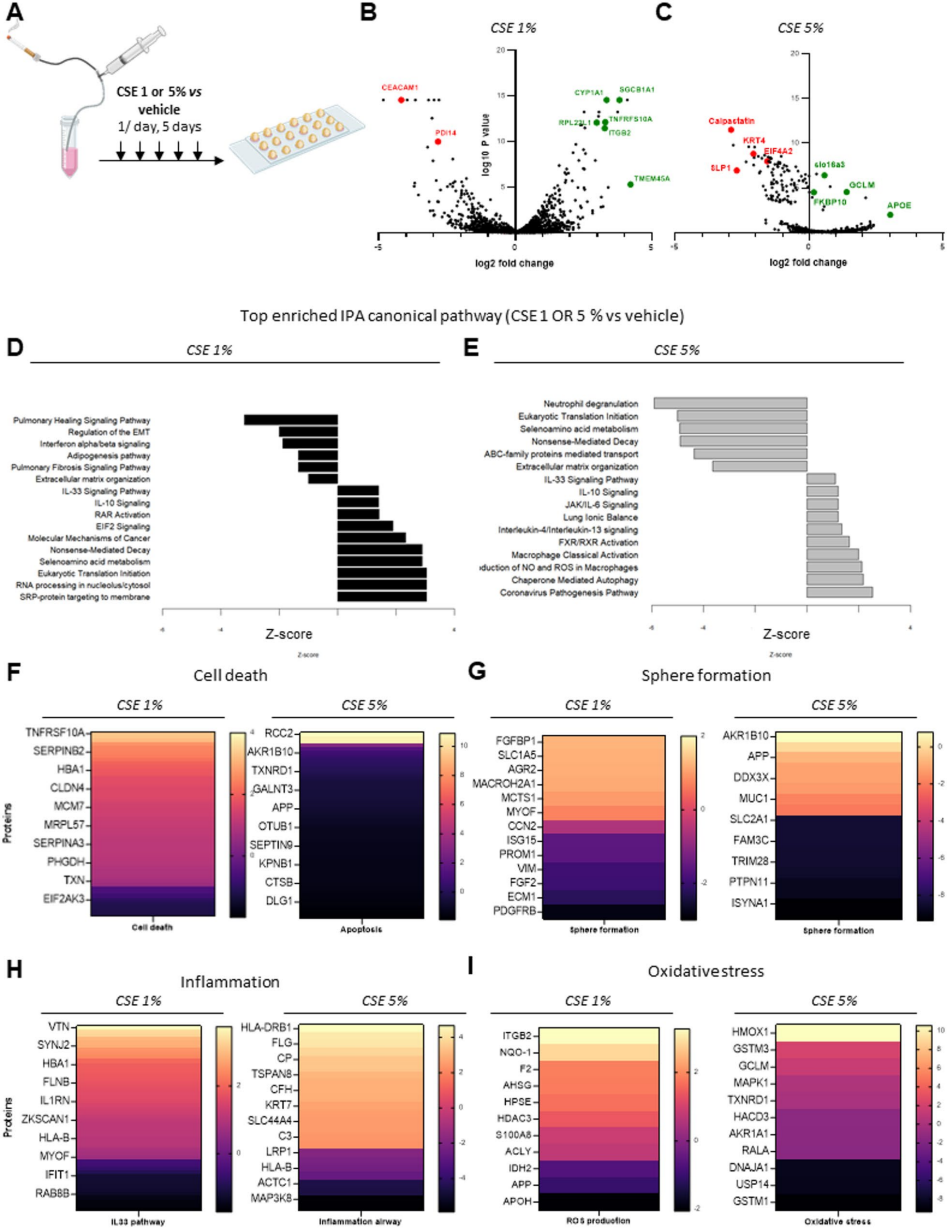
Chronic exposure to cigarette smoke extract (CSE) using 3D alveolospheres from non-COPD patients (**A**) 3D alveolospheres from non-COPD patients were exposed daily for 5 consecutive days to either 1% or 5% CSE. **B** Volcano plot displaying the differentially expressed proteins (DEPs) between the 1% CSE-treated group and the vehicle control (three donors). **C** Volcano plot displaying the DEPs between the 5% CSE-treated group and the vehicle control (four donors). **D** Bar graphs illustrating the top 16 enriched canonical pathways identified by Ingenuity Pathway Analysis (IPA) from the differentially expressed proteins (DEPs) in the 1% CSE group compared with the vehicle control (three donors). **E** Bar graphs illustrating the top 16 enriched canonical pathways identified by IPA from the DEPs in the 5% CSE group compared with the vehicle control (four donors). **F** Heatmap showing DEPs associated with cell death in alveolospheres exposed to 1% CSE versus vehicle and 5% CSE versus vehicle (orange: activation; purple: inhibition). **G** Heatmap showing DEPs associated with cell–cell interactions in alveolospheres exposed to 1% CSE, 5% CSE, and vehicle (orange: activation; purple: inhibition). **H** Heatmap showing DEPs associated with inflammation in alveolospheres exposed to 1% CSE, 5% CSE, and vehicle (orange: activation; purple: inhibition). **I** Heatmap showing DEPs associated with oxidative stress in alveolospheres exposed to 1% CSE, 5% CSE, and vehicle (orange: activation; purple: inhibition). COPD: chronic obstructive pulmonary disease; CSE: cigarette smoke extract; DEPS: differentially expressed proteins

Next, we assessed the ability of alveolospheres to accurately model pathophysiological features of chronic exposure to cigarette smoke. For this purpose, we exposed alveolospheres grown from non-COPD patients’ cells to 1% or 5% CSE for 5 consecutive days (Fig. 6A), then conducted proteomic analyses of differentially expressed proteins between the control and exposed groups. We identified 8,502 and 554 upregulated proteins, and 7,501 and 1,287 downregulated proteins, in the 1% and 5% CSE groups, respectively. Additionally, five pathways involved in emphysema—such as fatty acid metabolism, fibrogenesis, and cell death—were upregulated, whereas 10 protective pathways—such as cell survival and immune response—were downregulated after 5% CSE exposure (Supplementary Figure S8 A). CSE exposure led to the modulation of pathways known to be involved in the pathophysiology of emphysema, including cell death *via* apoptosis (Fig. 6D-F) and the disruption of cell-to-cell junctions (Fig. 6G, Supplementary Fig. 8D). These findings were confirmed by canonical pathway analyses, which indicated that pathways related to pulmonary healing and extracellular matrix organization were significantly downregulated after CSE exposure (Fig. 6D, E). In addition, quantitative image-based analysis of phalloidin-stained F-actin revealed that CSE exposure significantly reduced actin fiber length and induced cytoskeletal disorganization compared with vehicle (Supplementary Fig. 8C). Furthermore, CSE exposure significantly increased the expression of inflammatory markers such as TNF receptor and APOE (Fig. 6B, C) — known to activate the NLRP3 inflammasome in macrophages in lung diseases [22]. Between the top-enriched pathways, we identified virus pathogenesis and macrophages activation (Fig. 6D, E, Figure S8 E) particularly after 5% CSE exposure, and several interleukins pathways (IL4/IL13, IL10, IL33, Fig. 6H). In addition, CSE exposure also increased oxidative stress markers as shown via PDIA4 protein disulfide isomerase 4, calpastatin or CYP1A1 up-regulation (Fig. 6B, C), also known to be up-regulated in COPD AEC2 [23] and ROS production (Fig. 6I). Proteins associated with stemness or precursor state were also up-regulated, such as SGB1A1, KRT4, TMEM45a and Ribosomal protein L22 like 1 Fig. 6B, C), which was consistent with published data in organoids from COPD patients [24].

Thus, these findings show the increase of oxidative stress markers and proteins associated with stemness/precursor states, consistent with previous studies regarding organoids from COPD patients [24].

**Fig. 7:**
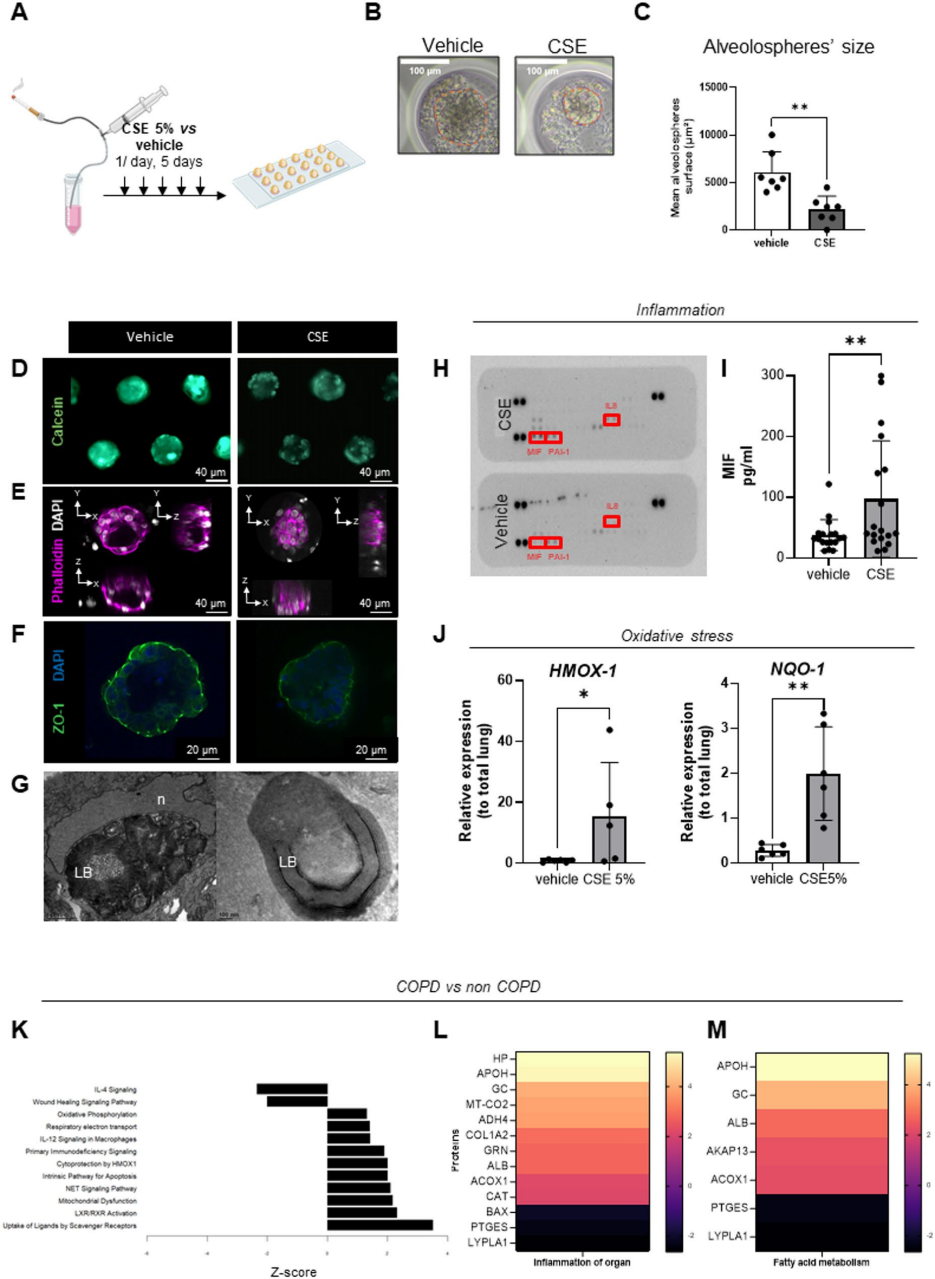
Chronic exposure to 5% cigarette smoke extract (CSE) using 3D alveolospheres. **A** Alveolospheres were exposed daily for 5 consecutive days to 5% CSE. **B** Representative image of an alveolosphere exposed to CSE versus vehicle. **C** Quantification of changes in organoid surface area after exposure to 5% CSE or vehicle, *n* = 7 patients. **D** Viability, as assessed by calcein staining, was reduced in alveolospheres exposed to 5% CSE compared with those exposed to vehicle. **E** Self-organization was disrupted in 5% CSE-exposed alveolospheres, as indicated by the absence of a central lumen (phalloidin in magenta). **F** 3D reconstruction of organoids stained for zona occludens-1 (ZO-1, green) and nuclei (DAPI, blue) exposed to 5% CSE, showing barrier dysfunction. **G** Lamellar bodies (LBs) appeared disorganized after 5% CSE exposure (n: nucleus). **H** Cytokine array analysis of supernatants from alveolospheres exposed to 5% CSE versus vehicle showed increased levels of MIF, PAI-1, and IL-8 (*n* = 2), indicating induced inflammation. **I** Enzyme-linked immunosorbent assay confirmed increased MIF levels (*n* = 18 patients). **J** qPCR analysis of HMOX-1 and NQO-1 indicated that 5% CSE induced oxidative stress (*n* = 6 patients). **K** Bar graphs illustrating the top 12 enriched canonical pathways identified by Ingenuity Pathway Analysis (IPA) from differentially expressed proteins in COPD (two donors) compared with non-COPD (2 donors). **L** Heatmap of differentially expressed proteins (DEPs) between 2 COPD and 2 non-COPD alveolospheres within the inflammation pathway (orange: activation; purple: inhibition). **M** Heatmap of DEPs between 2 COPD and 2 non-COPD alveolospheres within the fatty acid metabolism pathway (orange: activation; purple: inhibition). Data are presented as mean ± SEM. Statistical significance was assessed using non-parametric tests, * *p* = 0.05. ** *p* = 0.01.COPD: chronic obstructive pulmonary disease; CSE: cigarette smoke extract, HMOX-1: heme oxygenase-1; IL-8: interleukin-8; LB: lamellar bodies, MIF: migration inhibitory factor; n: nucleus, NQO-1: NAD(P)H dehydrogenase quinone 1; PAI-1: plasminogen activator inhibitor-1; ZO-1: zona occludens-1

Based on these results and previous data [25], we selected 5% CSE exposure for subsequent experiments (Fig. 7A) and exposed alveolospheres grown from 4 different patients cells. Compared with control condition, alveolospheres exposed to CSE were smaller (Fig. 7B, C), and calcein staining was weaker, indicating reduced cellular viability (Fig. 7D). Additionally, the architecture appeared disrupted, with the absence of a central lumen (Fig. 7E). The epithelial junctions were compromised with absence of intra-organoid ZO-1 staining (Fig. 7F) and minimal impaired adherens junctions (AJs) using TEM (Supplementary Figure S 8B); LBs appeared disorganized, enlarged, and clustered (Fig. 7G). Consistent with the proteomic analyses, alveolospheres grown from two patients showed increased levels of secreted inflammatory cytokines (MIF, IL-8, and PAI1, Fig. 7H), which was confirmed via enzyme-linked immunosorbent assay where MIF was also found to be significantly higher after CSE exposure (Fig. 7I). Moreover, the expression of oxidative stress–related genes (*HMOX1*, *NQO1*) was significantly increased, as indicated by RT-qPCR (Fig. 7J). Finally, proteomic analysis of alveolospheres derived from 2 COPD patients, compared with non-COPD ones, also exhibited increased expression of inflammatory and cell death markers (Fig. 7K), along with activation of inflammation and fatty acid metabolism pathways (Fig. 7L, M). Overall, the changes observed in alveolospheres derived from COPD lungs show a strong qualitative resemblance to those induced by CSE exposure. Indeed, Ingenuity Pathway Analysis revealed that most of the biological functions altered in the COPD versus non-COPD comparison were regulated in the same direction as in alveolospheres exposed to cigarette smoke extract compared with controls. In both settings, pathways related to cell death and lipid accumulation were activated, whereas signals associated with epithelial repair and cell viability were inhibited (Supplementary Figure S 8 F, G). These convergent signatures suggest that the COPD-associated alterations observed in alveolospheres partially recapitulate the molecular effects of cigarette smoke exposure, although direct quantitative comparisons were beyond the scope of the present study.

In summary, daily exposure of our standardized 3D alveolospheres to CSE 5% for 5 days reproduced key features of emphysema, including cell death, cell junction disruption, surfactant dysfunction, increased inflammation and oxidative stress, with functional alterations that mirror those observed in alveolospheres derived from COPD lungs.

## Discussion

The pathogenesis of emphysema involves impaired cellular junctions, surfactant dysregulation [7], chronic inflammation [26] and oxidative stress. Many cellular and molecular processes are difficult to study in animal models, and conventional 2D in vitro cultures fail to replicate key features of lung tissue. Consequently, in vitro 3D culture systems have emerged as valuable platforms for understanding pathological processes. Here, we developed a tunable, sustainable, and homogeneous 3D alveolosphere model derived from human primary AEC2 to investigate the mechanisms underlying emphysema. Compared to Matrigel-based spheroid cultures, alveolospheres generated in microwells display reduced heterogeneity in size. Using hydrogel-calibrated microwells, the model reproduced several essential aspects of native alveolar characteristics, including lumen formation, epithelial junctions development, and epithelial cell composition (AEC1 and AEC2) within alveolar-like structures. Furthermore, chronic CSE exposure induced key pathophysiological features of emphysema, such as increased cell death, cell junction disruption, oxidative stress, and inflammation. Finally, we successfully developed an innovative AI-driven tool to characterize alveolosphere maturation via SBF-TEM.

Our alveolar organoid model offers several advantages. Conventional methods for alveolosphere culture have suffered from low reproducibility and geometric heterogeneity [5, 8], limiting the scope and application of organoid research in respiratory diseases. In our model, AEC2 seeded into spherical microwells self-organized into spherical structures with progressive central lumen formation. Although the precise mechanisms of luminogenesis remain unclear in our model, key contributing factors have been shown to include curved geometry [27], fluid pressure [28], and actin polymerization followed by ion flux into the intracellular space and tight junction formation [29]. It has also been shown that an apical actin network, including tropomyosin and cytokeratin-based intermediate filaments, increases in size as the lumen expands [30]. Subsequently, tight junctions form, enabling lumen growth through the establishment of an osmotic pressure gradient [29]. By generating large numbers of organoids, our alveolosphere model could provide a high-throughput method for accurately studying the mechanisms underlying luminogenesis.

Second, our model is adjustable both in size and stiffness. We have achieved growing alveolospheres with a homogeneous size that closely resemble real human alveoli [19] and less variable than in Matrigel. Additionally, the stiffness of the hydrogel matrix can be modulated to mimic values reported for emphysema (~ 1 kPa) compared with native lungs (~ 5 kPa) [13–15]. Here, we evaluated the impact of varying hydrogel stiffness on organoid longevity and found that a stiffness of 5 kPa — closely matching the mechanical properties of native lung tissue—led to improved long-term maintenance of the organoids. In our study, stiffness directly impacted organoid formation, highlighting the critical role of matrix mechanics in supporting epithelial cell function and culture stability. This tunable feature of the system represents a promising avenue for future experiments. Indeed, the impact of matrix stiffness has received increasing interest in the context of emphysema, which is characterized by a decrease in elastic recoil [1]. At the microscale level, differentiated AEC1 cells, as well as their precursor AEC2 cells, are sensitive to mechanical stress and rigidity. Aberrant mechanical stretching and alterations in extracellular matrix stiffness, as observed in emphysema, can result in barrier dysfunction, metabolic dysregulation, cytotoxicity, and inflammation. In emphysema, the mechanical forces of normal breathing [31] contribute to inflammation-related [32] or elastase-induced [33] remodeling of the septal wall, leading to weakened collagen, elastin, and the entire alveolar structure [34, 35]. Furthermore, we confirmed that cigarette smoke disrupts epithelial integrity by altering epithelial cell-cell adhesion and inducing actin cytoskeleton remodeling, characterized by reduced actin fiber length and increased disorganization [36]. Intriguingly, de Hilster et al. [13] developed an in vivo hydrogel directly derived from decellularized human lung tissue—reduced to a powder and reconstituted—but the model failed to reproduce the low stiffness associated with emphysema. Overall, many unanswered questions remain regarding the micromechanics of the alveolus, particularly in the context of emphysema. Therefore, our tunable model may be especially valuable for mimicking this important biomechanical property of emphysematous lungs.

The expansion of AEC2 cells in 2D culture has previously been described in the literature, notably by Barkauskas et al., who demonstrated that primary AEC2 cells can be maintained and expanded in vitro under specific conditions [26]. However, we acknowledge that this 2D expansion of AEC2 cells may potentially induce transitional states [37], nevertheless here we provide evidence that the shortly expanded cells retained key features of the AEC2 phenotype, with no evidence of transition towards AEC1 prior to 3D culture. Specifically, the cells predominantly expressed surfactant protein C, a hallmark marker of AEC2 cells, and were positive for Lysotracker, indicating active lamellar bodies typical of functional AEC2 cells. Importantly, they were also negative for podoplanin and HTI-56, which are markers of AEC1. Therefore, these phenotypic analyses indicate that this effect is likely minimal in our system and unlikely to confound downstream 3D culture or disease modeling outcomes.

The growth of 3D-cultured alveoli may depend on cocultured fibroblasts, suggesting a crucial role for fibroblast-derived niche factors in maintaining the alveolar epithelium [23, 33]. Yao et al. [34] demonstrated the essential role of fibroblasts in mediating alveolar regeneration in air–liquid interface cultures. In contrast, we successfully maintained alveolospheres in culture for 14 days without a fibroblast niche, achieved through the use of optimized culture medium and hydrogel substrates. Thus, by leveraging the specific properties of our hydrogel and available commercial media, we describe a robust method for isolating and 3D-culturing human AEC2 cells without the need for feeder cells support. Future studies should investigate whether incorporating fibroblasts into our model could improve long-term maintenance and further differentiation.

Long-term maintenance remains a substantial challenge. Previously unknown intermediate cell state has been identified during the differentiation of AEC2 into AEC1 cells, particularly after lung injury. Several molecular markers, including KRT-8 and claudin-4, have been identified in this transient state, whereas the expression of canonical AEC1 and AEC2 markers is low [37, 38]. Our results suggest the presence of these intermediate cells in our alveolospheres, evidenced by the expression of claudin-4, and KRT-8 highlighting an ongoing dynamic differentiation process within our model. Furthermore, our AI-driven analyses of TEM data suggest the presence of AEC1 cells, identified by the absence of lamellar bodies (LBs), underscoring the robustness of this standardized tool in revealing key features of mature alveoli in vivo. Therefore, our data indicate that these 3D culture models offer a valuable approach for studying the mechanisms underlying alveolar pathology. It is well established that repeated insults, such as cigarette smoke exposure, lead to chronic inflammation and oxidative stress both in vivo and in vitro [25, 39]. Chan et al. [38] established and characterized COPD lung organoids derived from nasopharyngeal and bronchial human cells, successfully recapitulating in vivo differences between non-diseased and COPD organoids. However, they did not examine alveolar cells. Moreover, we observed an increase in proteins classically expressed in AEC2 progenitor cells, such as SCGB1A1 and TMEM45A, which have been implicated in COPD [23, 24, 40, 41]. Notably, SCGB1A1 was recently identified in the AEC2 cells of alveoli, particularly in COPD lungs [42], and TMEM45A is a marker of rbAEC2 progenitor cells whose expression is increased in emphysematous lungs [24] Additionally, the inflammatory process secondary to CSE exposure led to MIF secretion, consistent with previous findings in a mouse model of COPD [43]. In addition to the dynamic differentiation observed within our alveolospheres, we also noted alterations in organoid size following chronic CSE exposure. These changes may be partly driven by increased apoptosis or reduced proliferative capacity of alveolar progenitor cells, potentially linked to cellular senescence [44, 45]. Such effects are consistent with the well-established impact of repeated insults, such as cigarette smoke, on alveolar stem cell function, chronic inflammation, and oxidative stress both in vivo and in vitro. Taken together, these findings further support the relevance of our 3D culture model for studying the cellular and molecular pathways underlying alveolar pathology and emphysema.

This study had several limitations. First, the generated alveolospheres did not fully occupy the microwells. However, their size remains stable over time and exhibits minimal variability across wells. This represents a significant advantage over Matrigel-based systems, in which alveolospheres show high heterogeneity in size and shape, and which often suffer from batch-to-batch variability due to their complex and undefined composition [8, 10]. Second, although the presence of LBs at various maturation stages suggests surfactant secretion into the alveolosphere lumen, the specific components of this lumen could not be explored or directly exposed to CSE in our model. Indeed, apical surface access remains challenging due to its enclosure within the alveolosphere. However, future experiments aimed at reversing alveolosphere polarity — while maintaining appropriate barrier function — may be feasible by modifying substrate stiffness, as previously described for enteroids [46]. Such modulation could be performed using our hydrogel microstructuring method [12]. We also acknowledge that the system primarily recapitulates the early cellular and molecular events associated with chronic cigarette smoke exposure, rather than the complete architectural remodeling that defines emphysema. However, the model still provides a valuable platform for assessing early disease-related injuries and cellular dysfunctions that precede overt architectural destruction in emphysema. In addition, although CSE exposure is short-term, this model effectively captures early cellular events and subacute changes that precede the chronic remodeling characteristic of emphysema. Finally, although the presence of microvilli and tight junctions strongly indicates cell orientation, the definitive polarity of the cells remains to be established. Nevertheless, this model offers a standardized platform for elucidating pathways involved in emphysema and provides insights into potential therapeutic interventions.

## Conclusion

We developed a standardized 3D model using primary cells, evidencing key features of native alveoli such as presence of a lumen, surfactant production, epithelial junctions and presence of AEC2 and AEC1. Exposure to CSE for 5 days resulted in decreased cell viability, architectural disorganization, and increased oxidative stress and inflammatory markers. This model provides a valuable tool for investigating the mechanisms underlying emphysema.

## Supplementary Information

Supplementary Material 1.

Supplementary Material 2.

Supplementary Material 3.

## Data Availability

The datasets used and analyzed during the current study are available from the corresponding author upon reasonable request.

## Abbreviations

2D: Two-dimensional
3D: Three-dimensional
ABCA3: ATP-binding cassette subfamily A member 3
AJs: Adherens junctions
AEC1: Type I alveolar epithelial cells
AEC2: Type II alveolar epithelial cells
AI: Artificial intelligence
BMI: Body mass index
CSE: Cigarette smoke extract
COPD: Chronic obstructive pulmonary disease
CLDN4: Claudin 4
D: Day
DAPI: 4′,6-diamidino-2-phenylindole
DEPs: Differentially expressed proteins
DLCO: Diffusing capacity of carbon monoxide in the lung
FEV1: Forced expiratory volume in 1 s
FVC: Forced vital capacity
HE: Hematoxylin–eosin
HMOX-1: Heme oxygenase-1
HTII-280: Human type II cell
HU: Hounsfield units
KRT: Keratin
IL-8: Interleukin-8
IPA: Ingenuity Pathway Analysis
LAA%: Low attenuation area
LB: Lamellar bodies
MIF: Migration inhibitory factor
MV: Microvillosity
NQO-1: NAD(P)H dehydrogenase quinone 1
P2XR4: Purinergic receptor P2 × 4
PAI-1: Plasminogen activator inhibitor-1
PaO_2_: Partial pressure of oxygen in arterial blood
PDPN: Podoplanin
PDMS: Polydimethylsiloxane
PSCs: Pluripotent stem cells
RV: Residual volume, with
SBF: Serial Block-Face
SF: Surfactant
SFTP: Surfactant proteinSGB: secretoglobin
TEM: Transmission electron microscopy
TJs: Tight junctions
TLC: Total lung capacity
TTF-1: Thyroid transcription factor 1
UV: Ultraviolet
ZO-1: Zona occludens-1
