# Supplementary material for: A new tunable 3D alveolospheres model from human alveolar epithelial type 2 cells (AEC2) with reduced heterogeneity for studying cigarette smoke extract exposure
Source: Respir Res. 2026 Mar 17;27:179. doi: 10.1186/s12931-026-03628-z (PMC13107669; doi:10.1186/s12931-026-03628-z)
Supplement: Supplementary file 2 — Supplementary Material 2. [file 12931_2026_3628_MOESM2_ESM.pdf]

suppl

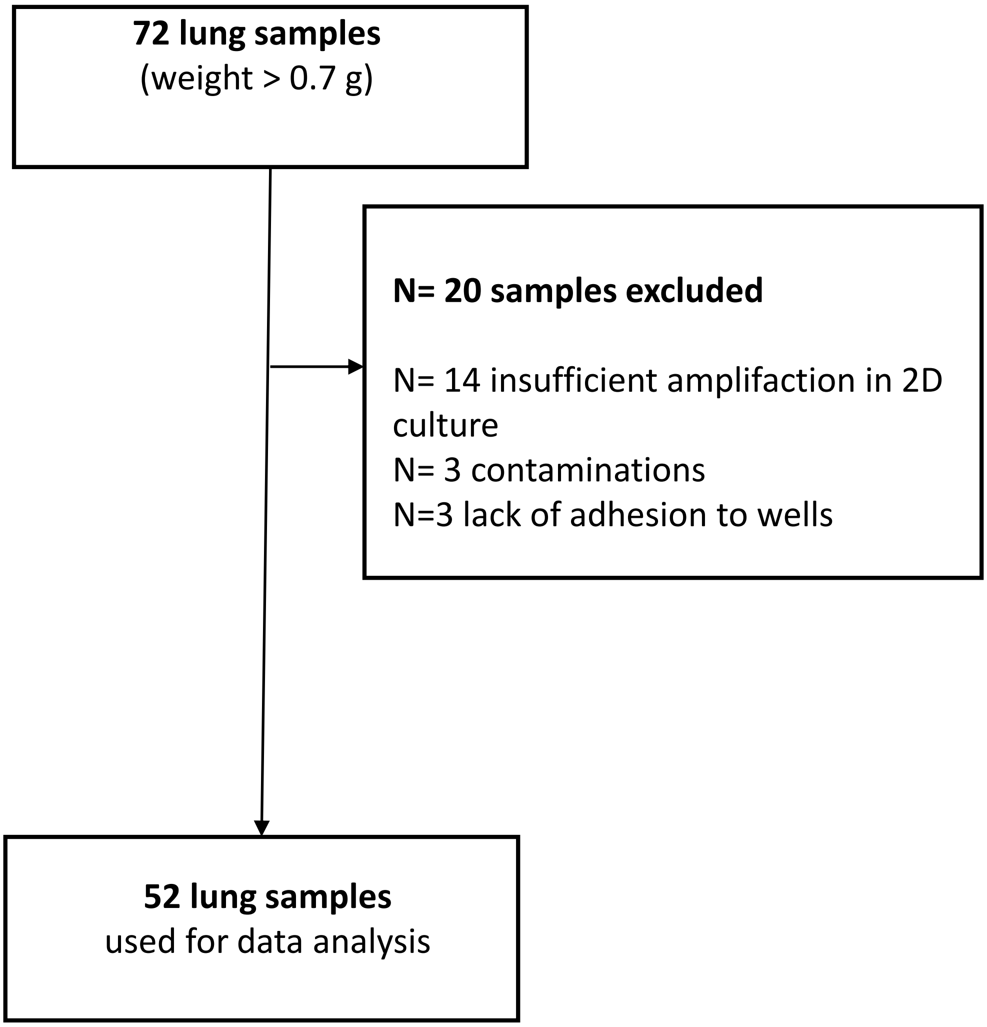
72 lung samples
(weight > 0.7 g)
N= 20 samples excluded
N= 14 insufficient amplifaction in 2D culture
N= 3 contaminations
N=3 lack of adhesion to wells
52 lung samples
used for data analysis

Figure S1: Flow chart

2D: 2 dimension, g: gram

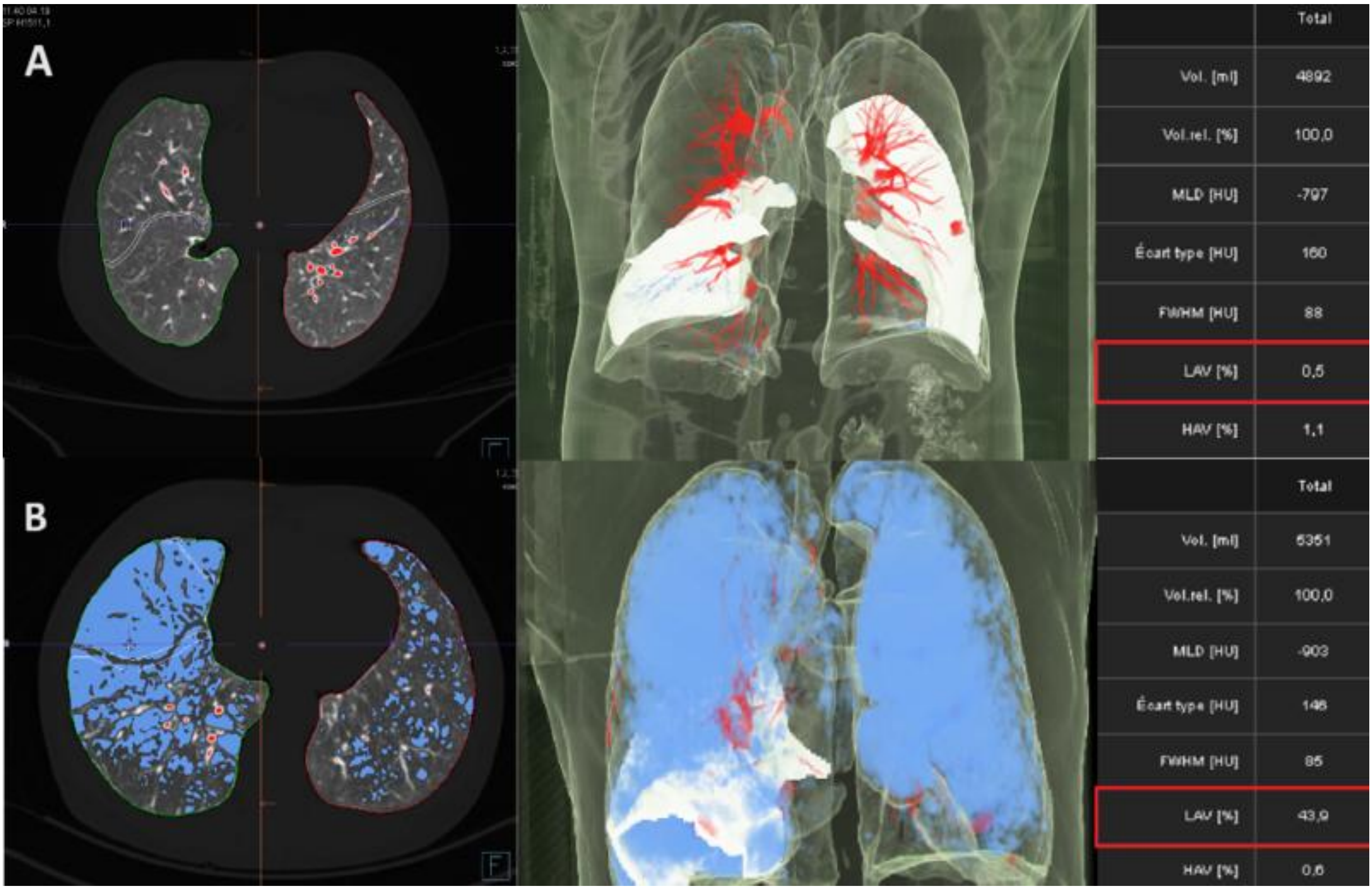

A
Total
Vol. [ml] 4892
Vol.rel. [%] 100,0
MLD [HU] -797
Écart type [HU] 160
FWHM [HU] 88
LAV [%] 0,5
HAV [%] 1,1
B
Total
Vol. [ml] 5351
Vol.rel. [%] 100,0
MLD [HU] -903
Écart type [HU] 146
FWHM [HU] 85
LAV [%] 43,9
HAV [%] 0,6

Figure S2: Illustration of 3D lung reconstruction and emphysema quantification on chest computed tomography scan using Syngovia© software. A) A non-emphysematous patient (LAA 0.5%) and B) A patient with severe pulmonary emphysema (LAV 43.9%).

FWHM: Full Width at Half Maximum, HAV: Hounsfield Attenuation Value, LAV: Low Attenuation Volume percentage, MLD: Mean Lung Density

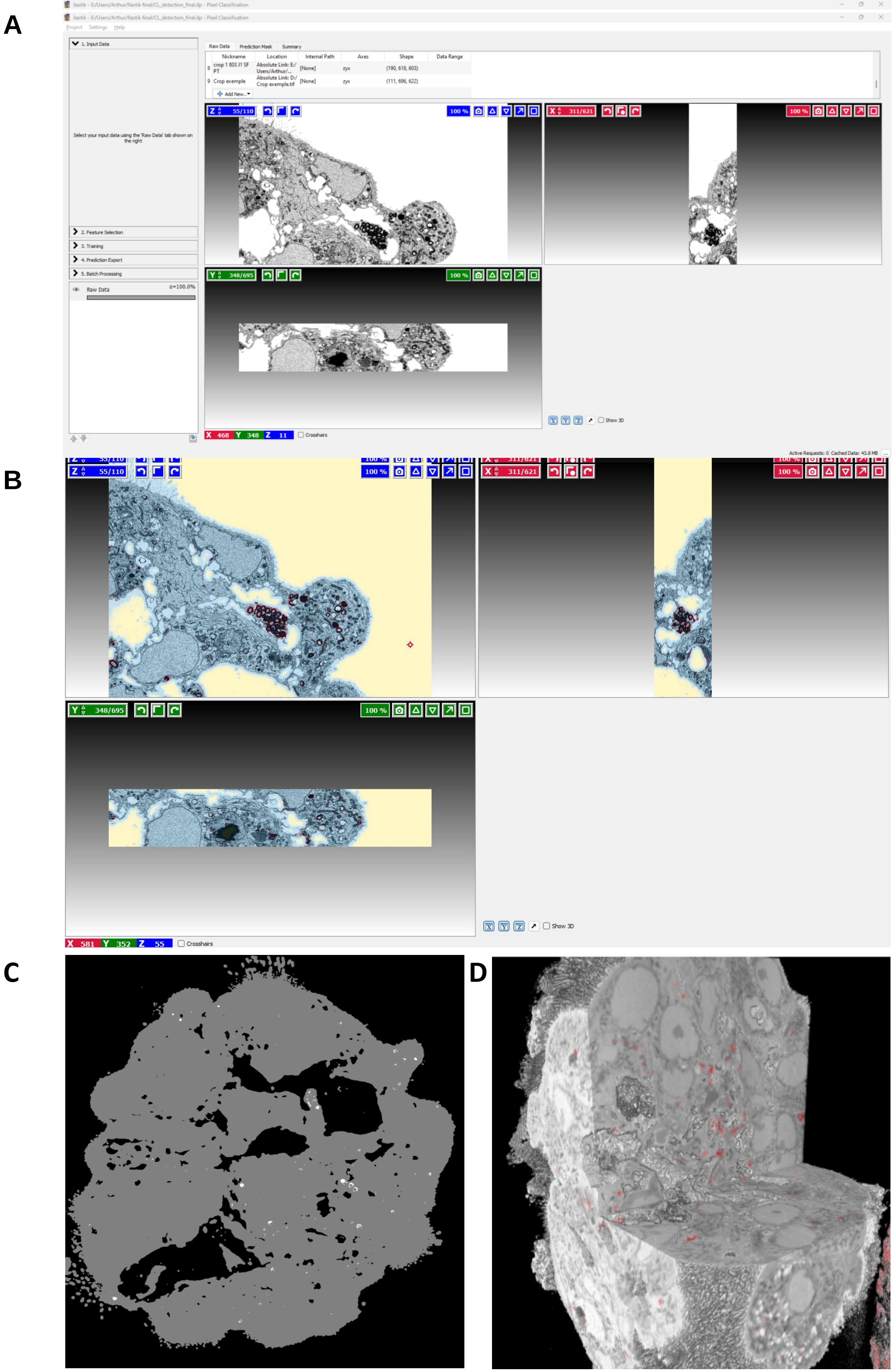
A
B
C
D
1. Input Data
Raw Data
Prediction Mask
Summary
Nickname
Location
Internal Path
Axes
Shape
Data Range
crop 1 803 J1 SF PT
Crop exemple
Add New...
Select your input data using the 'Raw Data' tab shown on the right
2. Feature Selection
3. Training
4. Prediction Export
5. Batch Processing
Raw Data
Crosshairs
Show 3D

Figure S3: **Lamellar bodies (LB) quantification by artificial intelligence (AI) with transmission electron microscopy (TEM) serial block face (SBF) images**
The processing of electron microscopy images to classify organoid cells based on their lamellar body (LB) content was performed following a three-step protocol: training the Ilastik model, training the Cellpose model, and analyzing lamellar body density.

(A) Crops in three, tagging the following structures: "Background," "Cell," and "Lamellar Bodies"
(B) Recognition of lamellar bodies automatically after training cell pose©.
(C) The final mask obtained with the complete stack.
(D) 2-D reconstruction,
(E) 3-D reconstruction of serial bloc face scanning electron microscopy (SBF-SEM) with artificial intelligence lamellar bodies recognition

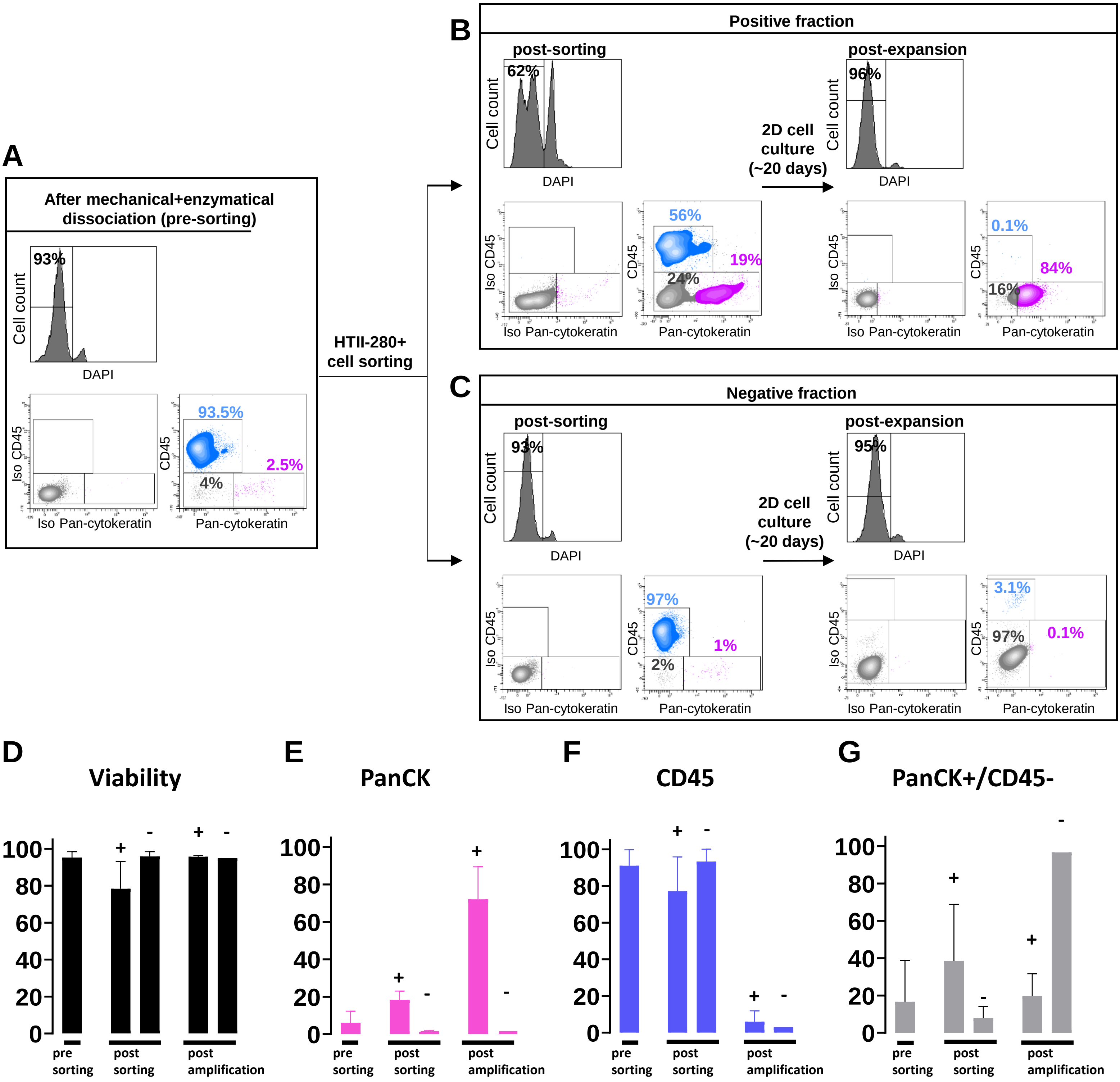
A
After mechanical+enzymatical dissociation (pre-sorting)
93%
Cell count
DAPI
Iso CD45
Iso Pan-cytokeratin
CD45
93.5%
2.5%
4%
Pan-cytokeratin
HTII-280+ cell sorting
B
Positive fraction
post-sorting
62%
post-expansion
96%
2D cell culture (~20 days)
56%
19%
24%
0.1%
84%
16%
C
Negative fraction
post-sorting
93%
post-expansion
95%
97%
1%
2%
3.1%
97%
0.1%
D
Viability
E
PanCK
F
CD45
G
PanCK+/CD45-
pre sorting
post sorting
post amplification

Figure S4: **2D culture characterization by flow cytometry, before microwells seeding**

(A) Flow cytometry after mechanical and enzymatical dissociation (pre-sorting) showing 93% viable cells, 93.5% hematopoietic cells and 2.5% epithelial cells

(B) Flow cytometry after HTII-280 + cell sorting showing 62% viable cells, 56% hematopoietic cells and 19% epithelial cells increasing to 84% after 20 days expansion in 2D culture condition

(C) Flow cytometry after HTII-280 - cell sorting showing 93% viable cells, 97% hematopoietic cells and 1% epithelial cells

(D) Viability of cells after mechanical and enzymatical dissociation, after HTII-280 cell sorting and after 20 days expansion in 2D culture condition (n=3)

(E) Proportion of epithelial cells (panCK+) after mechanical and enzymatical dissociation, after HTII-280 cell sorting and after 20 days expansion in 2D culture condition (n=3)

(F) Proportion of hematopoietic cells (CD45+) after mechanical and enzymatical dissociation, after HTII-280 cell sorting and after 20 days in 2D culture condition (n=3)

(G) Proportion of non epithelial non mononuclear cells (panCK-CD45-) after mechanical and enzymatical dissociation, after HTII-280 cell sorting and after 20 days expansion in 2D culture condition (n=3)

Data are presented as mean ± SEM from n = 3 biological replicates. Statistical significance was assessed using non-parametric tests, with significance defined as $p < 0.05$.

2D: 2 dimension, CD: cluster differentiation, DAPI: 4',6-diamidino-2-phenylindole, HTII-280: Human type 2 cells- 280kDa protein, panCK: pancytokeratin

2D culture characterization, HT2-280 positive fraction, post expansion, before microwells seeding

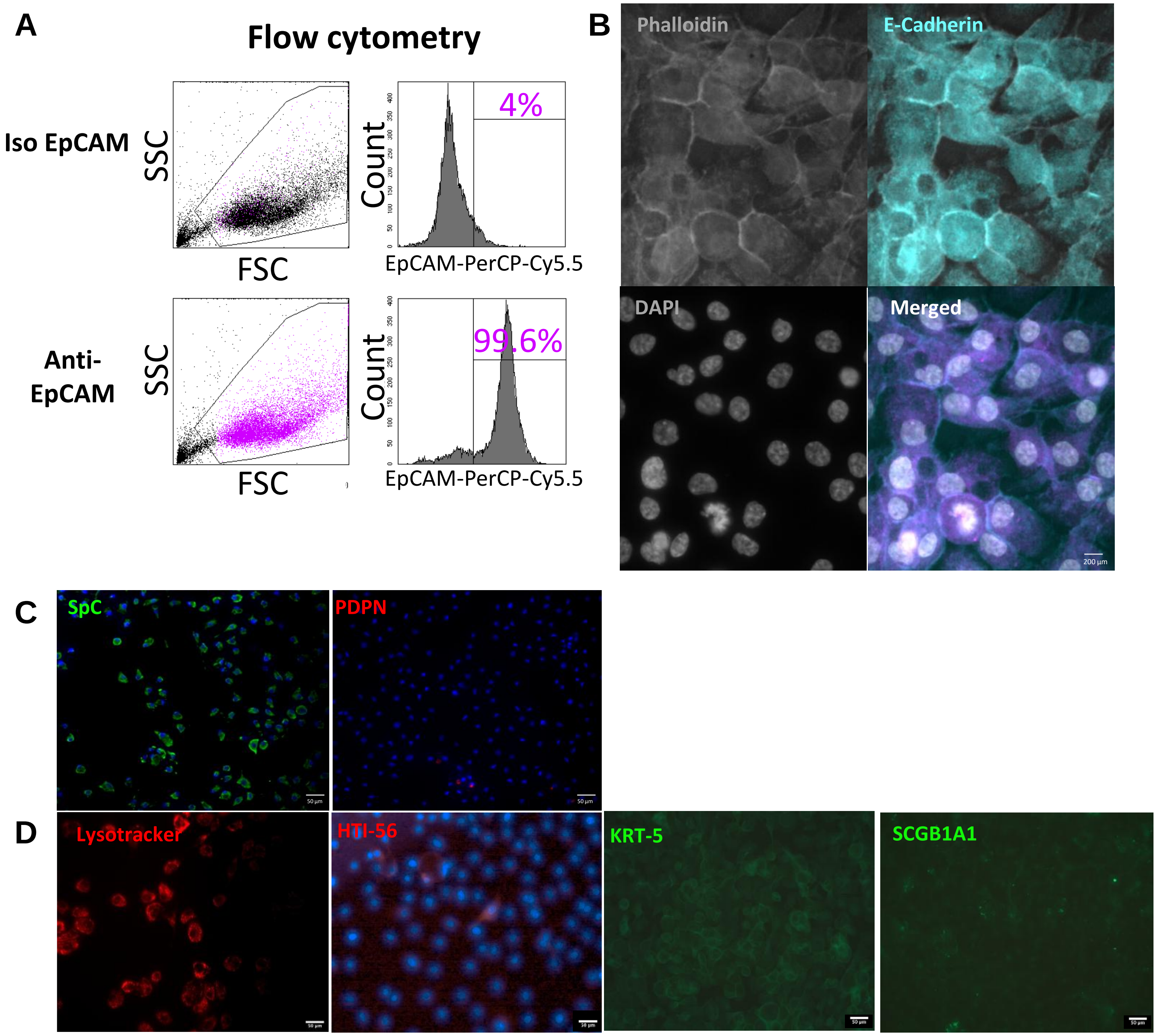

2D culture characterization, HT2-280 negative fraction, before microwells seeding at day 1

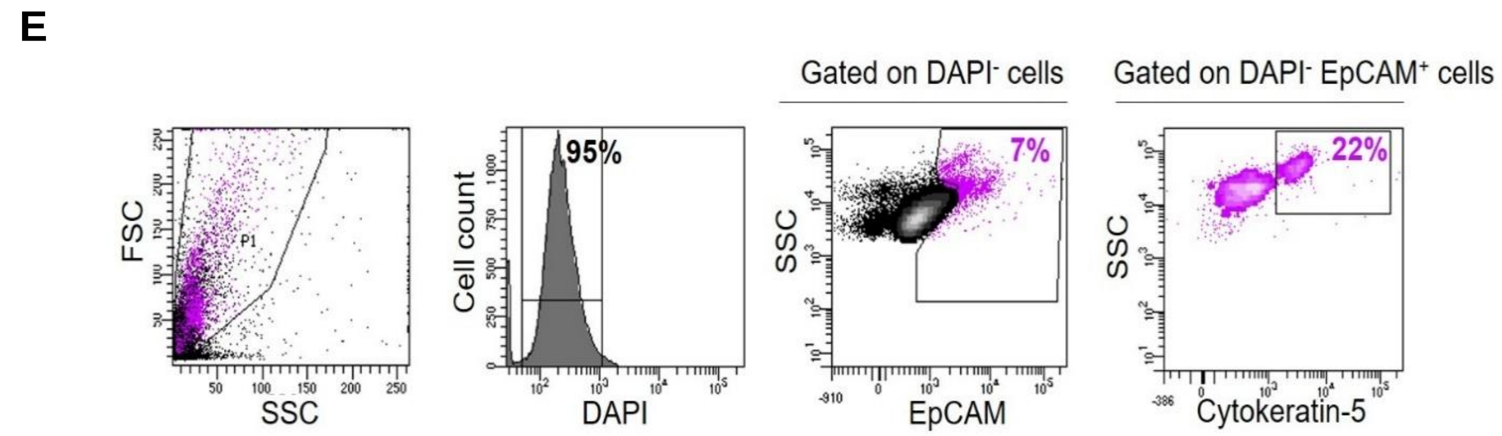

Figure S5: **2D culture characterization, post expansion, before microwells seeding (~ 20+/-5 days after 2D seeding)**

(A) From the HT2-280 positive fraction, flow cytometry showing 99% of epithelial phenotype (epCAM +) 20 days after 2D seeding

(B) From the HT2-280 positive fraction, fluorescent image of 2D-culture stained for cystoskeleton (phalloidin, grey), epithelial junction (E-cadherin, blue) and nuclei (DAPI, white) confirming epithelial phenotype

(C) From the HT2-280 positive fraction, fluorescent image of 2D-culture stained for AEC2 (Spc, green), AEC1 (podoplanin, red) and nuclei (DAPI, blue) confirming a majority of AEC2

(D) From the HT2-280 positive fraction, fluorescent image of 2D-culture stained for AEC2 (lysotracker, red), AEC1 (HTI-56, red), KRT-5, SCG1A1 confirming a majority of AEC2

(E) Flow cytometry characterization of the HTII-280 negative fraction. Cells were first selected based on FSC/SSC parameters (P1) and viability (DAPI negative, 95%). Among viable cells, EpCAM-positive epithelial cells represented approximately 7% of the HTII-280 negative fraction. Within the $EpCAM^{+}$ population, 22% of cells expressed cytokeratin-5.

2-D: 2 dimension, AEC1: alveolar type 1 cell, AEC2: alveolar type 2 cell, DAPI: 4',6-diamidino-2-phenylindole, EpCAM: Epithelial cell adhesion molecule, HTI-56: Human type 1 cells- 56kDa protein, KRT-5: Keratin 5, KRT-8: Keratin 8, SCG1A1: secretoglobin 1A1, PDPN: podoplanin, SPC: surfactant protein C

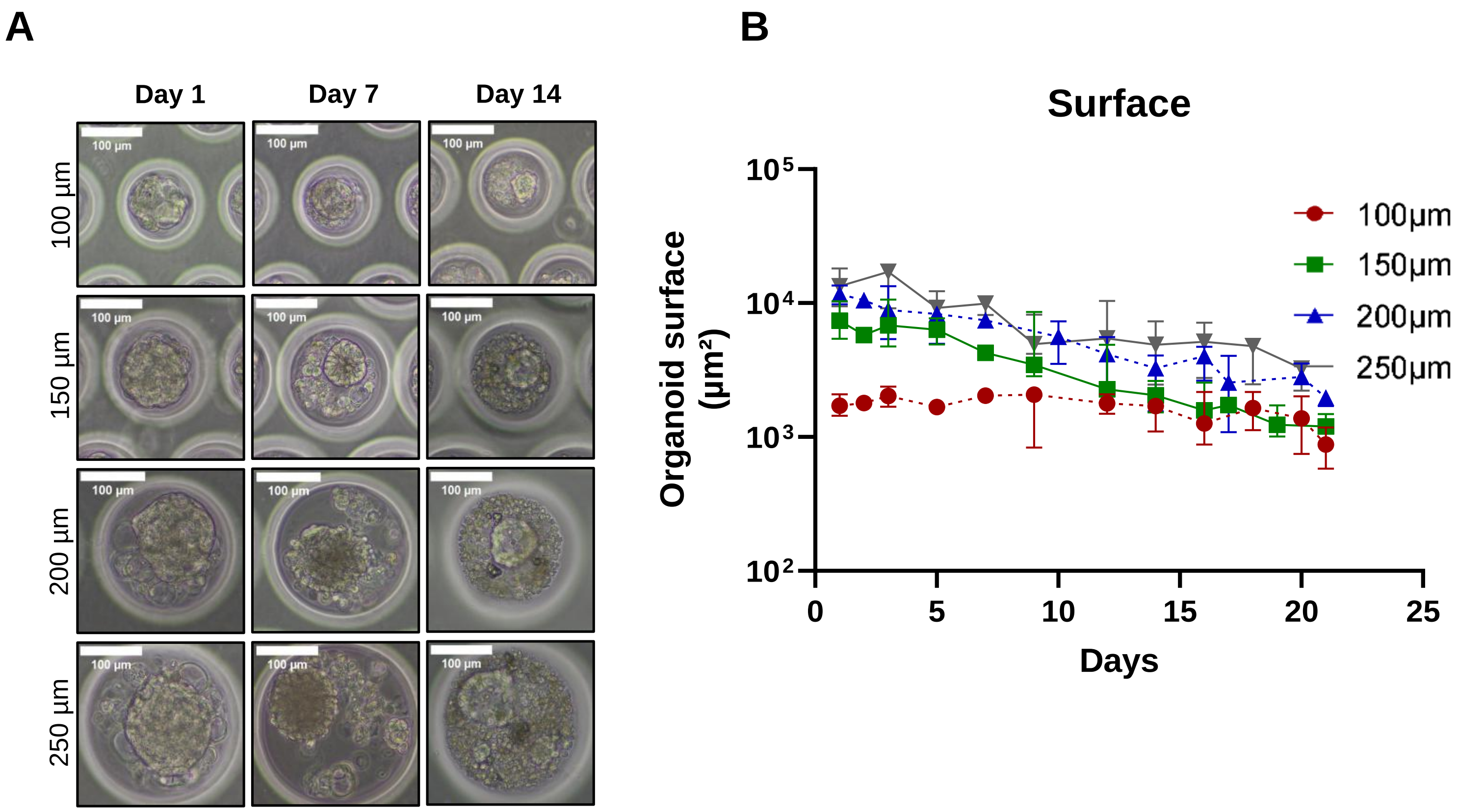
A
Day 1
Day 7
Day 14
100 µm
150 µm
200 µm
250 µm
100 µm
B
Surface
Organoid surface (µm²)
Days
100µm
150µm
200µm
250µm

Figure S6: 3D alveolosphere model from human type 2 alveolar epithelial cells.

(A) Alveolosphere maintained in culture until D14 in different diameters (100, 150, 200 and 250µm) of microwells.

(B) Alveolospheres surface until D14 according to different diameters (100, 150, 200 and 250µm). Dotted lines relate to data already shown in Figure 1G. Data are presented as mean ± SEM from *n* = 3 biological replicates.

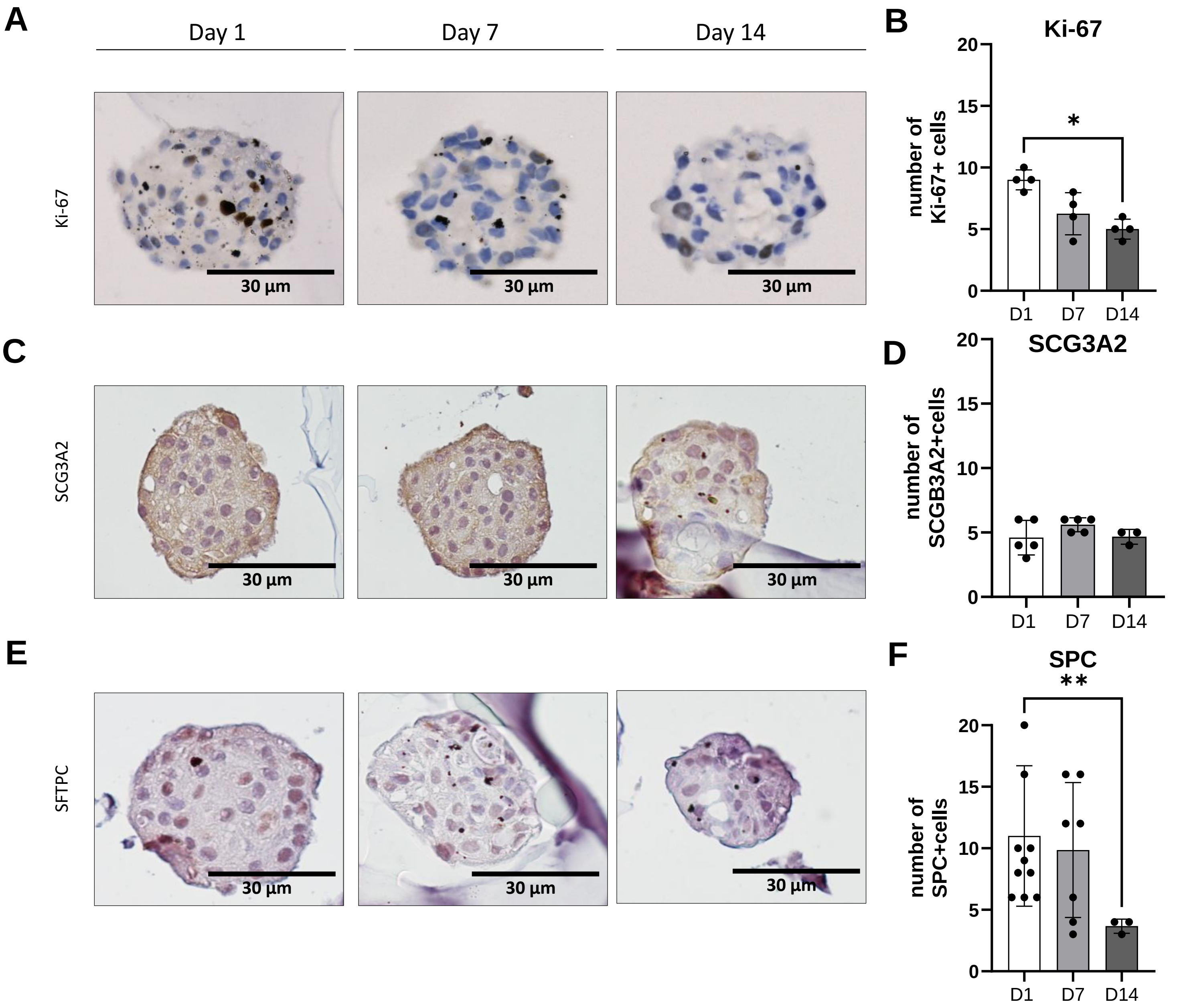

A
Day 1
Day 7
Day 14
Ki-67
30 µm
30 µm
30 µm
B
Ki-67
number of Ki-67+ cells
*
D1
D7
D14
C
SCG3A2
30 µm
30 µm
30 µm
D
SCG3A2
number of SCGB3A2+cells
D1
D7
D14
E
SFTPC
30 µm
30 µm
30 µm
F
SPC
**
number of SPC+cells
D1
D7
D14

Figure S7: Characterization of cellular differentiation in the 3D alveolosphere model.

(A) Bright-field images of HE staining and Ki-67 immunostaining. One representative paraffin-embedded tissue section was assessed per donor for each staining.

(B) Quantification from 4 to 6 patients at day (D) 1, 7 and 14, for Ki-67. Data are presented as mean ± SEM. Statistical significance was assessed using non-parametric tests, * p=0.05.

(C) Bright-field images of HE staining and SCG3A2 immunostaining. One representative paraffin-embedded tissue section was assessed per donor for each staining.

(D) Quantification from 4 to 6 patients at day (D) 1, 7 and 14, for SCGB3A2. Data are presented as mean ± SEM. Statistical significance was assessed using non-parametric tests, * p=0.05.

(E) Bright-field images of HE staining and SFTPC immunostaining. One representative paraffin-embedded tissue section was assessed per donor for each staining.

(F) Quantification from 4 to 6 patients at day (D) 1, 7 and 14, SFTPC. Data are presented as mean ± SEM. Statistical significance was assessed using non-parametric tests, ** p=0.01

SCG3A2, secretoglobin 3A2, SFTPC: surfactant protein C

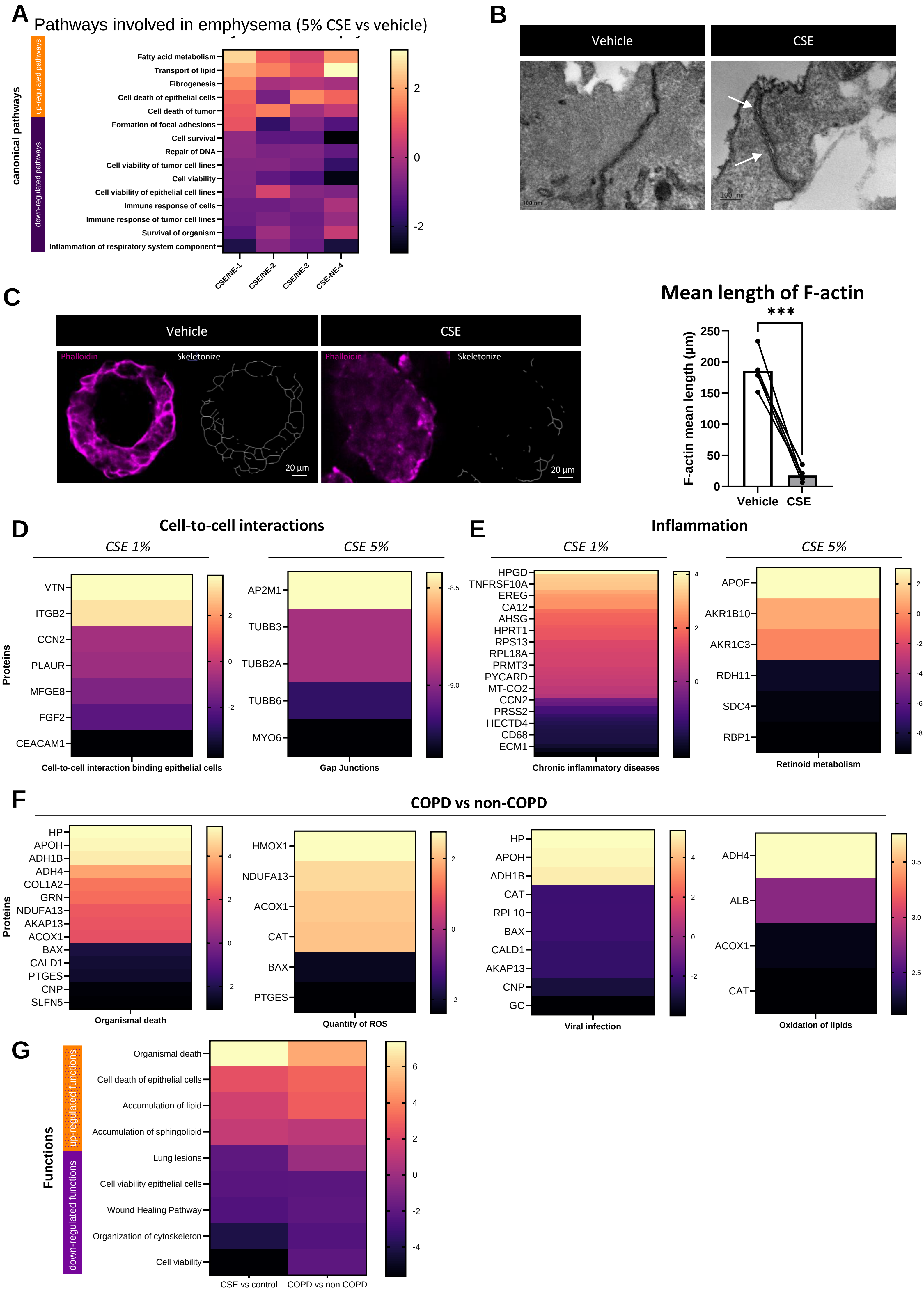
A
Pathways involved in emphysema (5% CSE vs vehicle)
canonical pathways
up-regulated pathways
down-regulated pathways
Fatty acid metabolism
Transport of lipid
Fibrogenesis
Cell death of epithelial cells
Cell death of tumor
Formation of focal adhesions
Cell survival
Repair of DNA
Cell viability of tumor cell lines
Cell viability
Cell viability of epithelial cell lines
Immune response of cells
Immune response of tumor cell lines
Survival of organism
Inflammation of respiratory system component
CSE/NE-1
CSE/NE-2
CSE/NE-3
CSE-NE-4
2
0
-2
B
Vehicle
CSE
100 nm
C
Vehicle
CSE
Phalloidin
Skeletonize
20 µm
Mean length of F-actin
***
F-actin mean length (µm)
250
200
150
100
50
0
Vehicle
CSE
D
Cell-to-cell interactions
CSE 1%
CSE 5%
Proteins
VTN
ITGB2
CCN2
PLAUR
MFGE8
FGF2
CEACAM1
Cell-to-cell interaction binding epithelial cells
AP2M1
TUBB3
TUBB2A
TUBB6
MYO6
Gap Junctions
-8.5
-9.0
E
Inflammation
CSE 1%
CSE 5%
HPGD
TNFRSF10A
EREG
CA12
AHSG
HPRT1
RPS13
RPL18A
PRMT3
PYCARD
MT-CO2
CCN2
PRSS2
HECTD4
CD68
ECM1
Chronic inflammatory diseases
APOE
AKR1B10
AKR1C3
RDH11
SDC4
RBP1
Retinoid metabolism
F
COPD vs non-COPD
Proteins
HP
APOH
ADH1B
ADH4
COL1A2
GRN
NDUFA13
AKAP13
ACOX1
BAX
CALD1
PTGES
CNP
SLFN5
Organismal death
HMOX1
NDUFA13
ACOX1
CAT
BAX
PTGES
Quantity of ROS
HP
APOH
ADH1B
CAT
RPL10
BAX
CALD1
AKAP13
CNP
GC
Viral infection
ADH4
ALB
ACOX1
CAT
Oxidation of lipids
3.5
3.0
2.5
G
Functions
up-regulated functions
down-regulated functions
Organismal death
Cell death of epithelial cells
Accumulation of lipid
Accumulation of sphingolipid
Lung lesions
Cell viability epithelial cells
Wound Healing Pathway
Organization of cytoskeleton
Cell viability
CSE vs control
COPD vs non COPD
6
4
2
0
-2
-4

Figure S8: ***Emphysema modelling in the 3D-alveolosphere model by exposure to 5% cigarette smoke extract (CSE)***

(A) Heatmap of the differentially expressed proteins between 5% CSE and vehicle exposed alveolospheres. Five up-regulated and ten down-regulated major pathways involved in emphysema were identified, based on IPA (orange: activation; purple: inhibition). Data are presented from *n* = 4 biological replicates.

(B) Electron microscopic image of embedded alveolospheres exposed to 5% CSE, showing impaired adherens junctions (AJs), as compared to vehicle exposed (white arrows).

(C) Representative images of phalloidin-stained F-actin (magenta) in alveolospheres cultured without CSE (vehicle) or 5% CSE. Image-based quantitative analysis shows a significant reduction in actin fiber length and increased cytoskeletal disorganization following CSE exposure (*n* = 5 biological replicates). Data are presented as mean ± SEM. Statistical significance was assessed using non-parametric paired-t-tests, ***$p < 0.001$.

(D) Heatmap of differentially expressed proteins between 1 and 5% CSE and vehicle exposed alveolospheres ; most of them being down-regulated (cell-to cell interaction and gap junction) (orange: activation; purple: inhibition). Data are presented from *n* = 4 biological replicates.

(E) Heatmap of differentially expressed proteins between 1 and 5% CSE and vehicle exposed alveolospheres; most of them being down-regulated (inflammation and retinoid metabolism) (orange: activation; purple: inhibition). Data are presented from *n* = 4 biological replicates.

(F) Heatmap of differentially expressed proteins of alveolospheres from 2 COPD and 2 non COPD patient; most of them being up-regulated (organismal death, quantity of ROS, oxidation of lipids) and some being down-regulated (viral infection) (orange: activation; purple: inhibition).

(G) Heatmap of the differentially expressed functions between 5% CSE and vehicle exposed alveolospheres and between COPD and non COPD patients. Four up-regulated and five down-regulated major functions involved in emphysema were identified, based on IPA (orange: activation; purple: inhibition). Data are presented from respectively *n* = 4 biological replicates and n= 2 replicates.

3-D: 3 dimension, *COPD: Chronic obstructive pulmonary disease, CSE: cigarette smoke extract, ROS: reactive oxygen species*
